# Non-canonical cleavage of GPR125/ADGRA3 releases extracellular receptor fragments detectable in cerebrospinal fluid

**DOI:** 10.1016/j.isci.2026.117551

**Published:** 2026-09-21

**Authors:** Anna L.S. Walser, Olav Larsen, Chiara Stüdle, Nicoline Wichmand Thorsen, Florian Seufert, Anna Katarzyna Drzazga, Renata Mężyk-Kopeć, Isha Raj, Filippa Liliendahl Qvist, Sofie Morsing Bagger, Gertrud Malene Hjortø, Trine Toft-Bertelsen, Emily Slowikowski, Silvia Bergt, Eva Ganseman, Christoffer Knak Goth, Juliane Lokau, Christoph Garbers, Hiroshi Ishikawa, Horst Schroten, Paul Proost, Anja Hviid Simonsen, Nicholas M.I. Taylor, Joanna Bereta, Nanna MacAulay, Peter W. Hildebrand, Niels Henning Skotte, Katja Spiess, Tobias Langenhan, Michael J. Davies, Britta Engelhardt, Pedro E. Marques, Mette M. Rosenkilde

**Affiliations:** 1 Laboratory of Molecular Pharmacology, Department of Biomedical Sciences, University of Copenhagen, 2200 Copenhagen N, Denmark; 2 Laboratory of Molecular Immunology, Department of Microbiology, Immunology and Transplantation, Rega Institute for Medical Research, KU Leuven, 3000 Leuven, Belgium; 3 Theodor Kocher Institute, University of Bern, 3012 Bern, Switzerland; 4 Department of Biomedical Sciences, Panum Institute, University of Copenhagen, 2200 Copenhagen N, Denmark; 5 Institute of Medical Physics and Biophysics, Medical Faculty, Leipzig University, 04103 Leipzig, Germany; 6 Faculty of Biochemistry, Biophysics and Biotechnology, the Jagiellonian University in Kraków, 30-387 Kraków, Poland; 7 Novo Nordisk Foundation Center for Protein Research, ICMM, Faculty of Health and Medical Sciences, University of Copenhagen, 2200 Copenhagen N, Denmark; 8 Department of Drug Design and Pharmacology, Faculty of Health and Medical Sciences, University of Copenhagen, 2100 Copenhagen Ø, Denmark; 9 Department of Neuroscience, University of Copenhagen, 2200 Copenhagen N, Denmark; 10 Institute of Clinical Biochemistry, Hannover Medical School, 30625 Hannover, Germany; 11 Laboratory of Clinical Regenerative Medicine, Department of Neurosurgery, University of Tsukuba, Tsukuba, Ibaraki 305-8575, Japan; 12 Pediatric Infectious Diseases, Department of Pediatrics, Medical Faculty Mannheim, Heidelberg University, 68167 Mannheim, Germany; 13 Danish Dementia Research Centre, Copenhagen University Hospital – Rigshospitalet, 2100 Copenhagen N, Denmark; 14 Rudolf Schӧnheimer Institute of Biochemistry, Division of General Biochemistry, Medical Faculty, Leipzig University, 04103 Leipzig, Germany; 15 Institute of Biology, Faculty of Life Sciences, Leipzig University, 04103 Leipzig, Germany; 16 Comprehensive Cancer Center Central Germany (CCCG), 04103 Leipzig, Germany

**Keywords:** GPR125, ADGRA3, adhesion G protein-coupled receptors, GPCR, cleavage, shedding, N-terminal fragment, cerebrospinal fluid

## Abstract

Adhesion G protein-coupled receptors (aGPCRs) are crucial for barrier tissues and typically autoproteolytically cleaved into N- and C-terminal fragments (NTF and CTF) that remain non-covalently associated until separation is triggered. Here, we show that human GPR125 (ADGRA3), expressed in choroid plexus epithelium, is not cleaved at the GPCR autoproteolysis site (GPS), consistent with its divergent SLS motif. Instead, it undergoes non-canonical proteolytic processing that generates and releases atypical, non-canonical N-terminal fragments (ncNTFs), a process further enhanced by insulin. Using mutational analyses, metalloprotease inhibition, and ADAM10/17-deficient cells, we find that multiple proteases likely participate in cleavage. Mass spectrometry reveals one specific cleavage site, and real-time imaging uncovers GPR125’s intracellular trafficking and shedding dynamics. Changing the GPS destabilizes the ncNTF-ncCTF association and increases ncNTF release, thereby promoting cell viability. Finally, detection of multiple GPR125-derived peptides in human cerebrospinal fluid suggests ncNTF shedding *in vivo* and possible biological relevance.

## Highlights

• GPR125 undergoes non-canonical proteolytic processing and shedding
• GPS mutations alter fragment release without preventing cleavage
• GPR125-derived N-terminal peptides are detected in human CSF
• Insulin enhances extracellular accumulation of GPR125 fragments

## Graphical abstract

**Figure:**
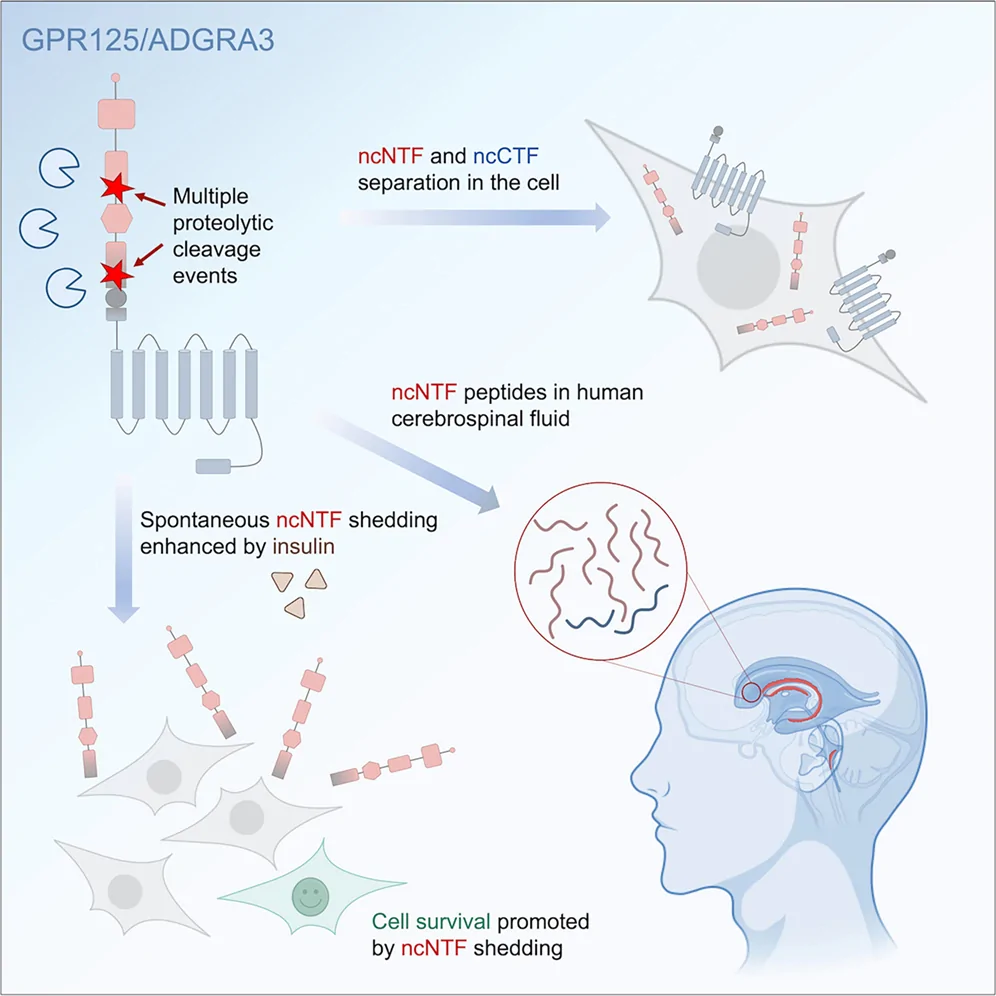

## Teaser

Natural sciences; Biological sciences; Neuroscience; Molecular neuroscience

## Introduction

Adhesion G protein-coupled receptors (aGPCRs) constitute a large family of GPCRs with 32 members in humans and are involved in various (patho-) physiological processes.^1^^,^^2^ Whereas ∼36% of FDA-approved drugs target GPCRs,^3^ there are no drugs targeting aGPCRs, primarily due to a lack of understanding of how individual members are functionally modulated and how this translates to their role in physiology.

GPR125/adhesion G protein-coupled receptor A3 (ADGRA3) is highly expressed in the secretory epithelium of the choroid plexus, where it is upregulated upon traumatic brain injury.^4^ Nonetheless, its role in the choroid plexus and the central nervous system (CNS) is largely unexplored. The epithelium of the choroid plexus is central to maintaining the brain’s internal environment, as it forms the blood-cerebrospinal fluid barrier.^5^ Moreover, the choroid plexus is the primary producer of cerebrospinal fluid and an important source of bioactive molecules involved in brain development, function, and repair. Hence, circulating cerebrospinal fluid serves as a key signaling route connecting distant regions of the CNS.^5^^,^^6^^,^^7^

GPR125 has diverse roles outside the CNS. It was originally identified as a spermatogonial progenitor marker, but it is also involved in the development of the mammary glands and urogenital tract.^8^^,^^9^^,^^10^^,^^11^ It has recently been implicated in adipose thermogenesis and amelioration of insulin resistance in mice, a role that may be mediated by its Gs-coupling.^12^^,^^13^ GPR125 is also associated with differentiation-specific signaling such as planar cell polarity and the recruitment of Discs large (Dlg), a tumor suppressor protein, and is correlated with both positive and negative outcomes in cancers.^10^^,^^14^^,^^15^ Although no endogenous ligands have been identified, GPR125 has been seen to constitutively internalize in endosomes in culture.^16^

On the structural side, aGPCR family members share key features, including a large extracellular N-terminal region with the GPCR-Autoproteolysis INducing (GAIN) domain directly N-terminal to the seven transmembrane (7TM) domain and intracellular region. Many aGPCRs undergo autoproteolytic processing at a three-amino-acid sequence HL|T/S (| denotes the cleavage site), known as the GPCR proteolysis site (GPS), located within the GAIN domain.^17^^,^^18^ Although GPR125 has a divergent SLS sequence at this position, it was recently proposed to undergo conventional autoproteolytic cleavage.^19^ The cleavage at the GPS, which divides the receptor into the N-terminal and C-terminal fragments (NTF and CTF), occurs N-terminal to a protein-internal tethered agonist sequence (Stachel), a conserved ∼7–20 amino acid sequence at the N-terminus of the CTF.^20^ In addition to the conventional autoproteolysis at the GPS, various proteases and alternative autoproteolytic cleavage sites have been reported or predicted for aGPCRs.^21^^,^^22^^,^^23^^,^^24^^,^^25^^,^^26^^,^^27^ For receptors that do not have the conventional autoproteolysis sequence at the GPS and thus are not cleaved by the conventional autoproteolysis at the GPS, the resulting receptor fragments are denoted non-canonical (nc) NTF and corresponding ncCTF.

The unique aGPCR structure allows for diverse paths to activation. In autoproteolytically cleaved aGPCRs, the NTF and CTF remain non-covalently bound,^28^^,^^29^^,^^30^ yet several aGPCRs are not self-cleaved.^31^^,^^32^ In the absence of self-cleavage, a direct interaction between the GAIN and 7TM domains may regulate receptor activation in a Stachel-independent mode.^33^^,^^34^^,^^35^ In contrast, receptors that self-cleave can signal in a Stachel-dependent manner, too.^36^ When the NTF is released at the GPS through adhesive ligand interactions and mechanical force, it can uncap the tethered agonist, and this uncapping induces full receptor activation.^37^^,^^38^^,^^39^ Mechanical force is often required for NTF release,^39^^,^^40^^,^^41^^,^^42^^,^^43^^,^^44^^,^^45^ although NTF and ncNTF shedding has also been reported without mechanical stimulation.^22^^,^^24^^,^^26^^,^^27^^,^^46^

Shed, soluble NTFs and ncNTFs have been shown to modulate important biological functions, including angiogenesis, tumorigenesis, cell proliferation, and survival.^26^^,^^27^^,^^39^^,^^47^^,^^48^^,^^49^ Identifying the different forms of aGPCRs and their regulatory processes is thus key to understanding translational applications and drug target potential.

This study demonstrates that GPR125 undergoes cleavage and releases its ncNTF, which we found to be enhanced by insulin stimulation. Moreover, the shed ncNTF improved cell viability *in vitro*. We show that cleavage does not occur at the GPS sequence, consistent with GPR125’s divergent SLS sequence, and that cleavage involves cellular metalloproteases, as revealed by mutational analyses, enzyme inhibition, and the use of ADAM10/17-deficient cells. Through mass spectrometry (MS) analysis of the shed GPR125 fragments, we detected multiple endogenous cleavage fragments from possible cleavage sites, of which we were able to identify one site by both neo-N-terminal and neo-C-terminal peptides. By real-time imaging, we reveal GPR125’s intracellular trafficking and shedding dynamics *in vitro*. We detect GPR125 peptides in human cerebrospinal fluid, suggesting that shedding may occur *in vivo*.

## Results

### GPR125 is cleaved near the GPS domain, and the N-terminal fragment is shed

GPR125 was previously reported to be autoproteolytically cleaved at its non-consensus GPS triad SL|S, dividing the receptor into an N- and C-terminal fragment, NTF and CTF.^19^ These fragments were presumed to form a stable, non-covalent complex given their demonstrated affinity for each other; however, the complex may also be transient. We used a plasmid vector encoding a double-tagged human (h) GPR125 construct with two V5 tags at the C-terminus and a FLAG tag at the N terminus (Figure 1A) to investigate the cleavage pattern. As a control, we included the human GPR125 homolog ADGRA2/GPR124 from the same aGPCR subfamily, which also has a non-consensus GPS and has been reported to be cleaved by several proteases.^23^^,^^43^

**Figure 1:**
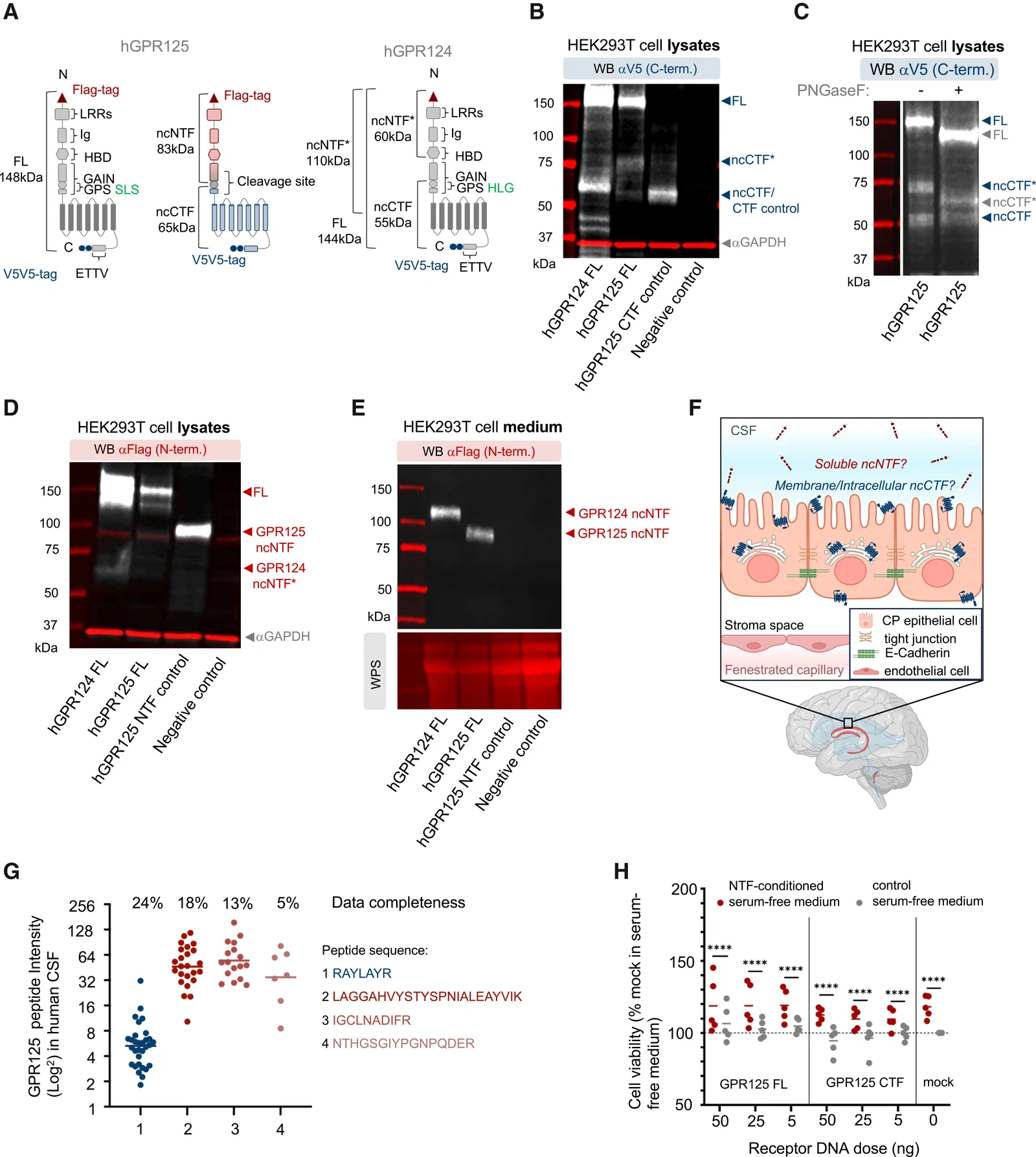
GPR125 peptides can be found in cerebrospinal fluid, and released ncNTF of GPR125 promotes cell survival *in vitro* (A) Schematic overview of hGPR125 and hGPR124 constructs indicating the size of the full-length (FL) receptor and non-canonical C- and N-terminal fragments (ncCTF and ncNTF). The GPS of hGPR125 is Ser-Leu-Ser (SLS). The GPR124 cleavage fragments shown are from.^26^^,^^47^ LRRs, leucine-rich repeats; Ig, immunoglobulin-like; HBD, hormone-binding domain; ETTV tetrapeptide Glu-Thr-Thr-Val, a class I PDZ domain-binding motif (PBM). (B) hGPR125 has two cleavage sites within its extracellular domain. (From the left) Immunoblot analysis of *HEK293T* cells expressing V5V5-tagged hGPR124 FL, hGPR125 FL, or hGPR125 ncCTF. Anti-V5 antibody was used to detect the C-terminal tag of hGPR124 or hGPR125. Anti-GAPDH was included as a loading control. The negative control shows cell lysates from cells expressing the empty vector (pcDNA3.1+). Arrows indicate the FL and cleavage fragments. (C) hGPR125 FL and ncCTF∗ bands are glycosylated. Immunoblot analysis of PNGase F-treated HEK293T cells expressing V5-tagged hGPR125 FL. The anti-V5 recognizes the C-terminal tag of hGPR125. Arrows indicate the FL and cleavage fragments in the samples treated with (gray arrows) and without (dark blue arrows) PNGase F. (D and E) hGPR125 sheds most of its ncNTF into the *HEK293T* cell medium. (D) (Left) Immunoblot analysis of HEK293T cell lysates with Flag-tagged proteins. The anti-Flag detects the N-terminal tag of hGPR125 and hGPR124. The negative control shows cell lysates from cells expressing the empty vector (pcDNA3.1+). Arrows indicate the FL and the cleavage fragments. Anti-GAPDH was included as a loading control. (E) Western blot analysis of Flag-stained HEK293T cell supernatant. A whole protein stain (WPS) was performed as a loading control. Arrows indicate the FL and the cleavage fragments. The hGPR124 cleavage forms confirm previous data by.^26^^,^^47^ (F) Schematic overview of the human choroid plexus (CP) and cerebrospinal fluid (CSF) shows the hypothesized expression of hGPR125. Figure created in part with BioRender.com. (G) Mass spectrometry analysis of human cerebrospinal fluid identified four distinct tryptic peptides derived from GPR125. (H) Viability of transiently transfected HEK293T cells expressing different amounts of either full-length (FL) or C-terminal fragment (CTF) of hGPR125 after 24-h incubation in serum-free medium with or without non-canonical N-terminal fragment (ncNTF) of hGPR125. Different receptor expression levels were obtained by applying 50, 25, 5, or 0 (mock) ng of receptor DNA per 30,000 cells. Data were collected from five independent experiments presented individually, in reference to the viability of mock-transfected cells incubated in serum-free medium without ncNTF. Statistically significant differences in viability were verified with two-way ANOVA and Fisher’s least significant difference (LSD) post-hoc test and depicted between ncNTF-stimulated and non-stimulated cells transfected with the same amount and type of receptor DNA, with ∗∗∗∗ for *p* < 0.0001. Full statistical details are provided in Table S4. See also Figures S1 and S2.

We expressed double-tagged hGPR125 and hGPR124 in HEK293T cells and analyzed the protein fragments’ sizes in cell lysates by western blotting with antibodies targeting the C-terminal V5-tags. For hGPR125, we observed two strong protein bands; one around 150 kDa corresponding to the full-length (FL) receptor and one around 65 kDa, which we termed ncCTF based on its electrophoretic mobility being comparable to that of a recombinant CTF construct encompassing the region from the +1 position of the predicted GPS to the C-terminus.^19^ We also observed a weaker band at around 80 kDa, which we termed ncCTF∗ (Figures 1A and 1B). For hGPR124, we observed a band at around 65–70 kDa, which we named ncCTF, and a band corresponding to the FL receptor (Figures 1A and 1B).

Removal of oligosaccharides via PNGase F treatment caused a shift of ∼25 kDa for the GPR125 FL band and ∼10 kDa for the ncCTF∗ band, but none for the ncCTF band (Figure 1C). Hence, our data suggest that the ncCTF bands could represent distinct cleavage products of GPR125 rather than differently N-glycosylated forms of the same protein species.

We next analyzed the level of ncNTF in the cell lysates using antibodies for the N-terminal FLAG tag. Consistent with the FL band detected from the C-terminus (Figure 1B), we observed a strong FL band; however, surprisingly, we detected only a very weak band at 83 kDa corresponding to the ncNTF (Figure 1D). Moreover, we did not detect an ncNTF∗ band corresponding to the ncCTF∗ at approximately 68 kDa. Therefore, we hypothesized that GPR125 releases most of its ncNTF and possibly ncNTF∗ into the cell medium.

Consistent with this, we found the GPR125 ncNTF in the cell medium similar to the corresponding ncNTF of the positive control hGPR124 (Figure 1E). However, we did not detect a fragment corresponding to the hypothesized ncNTF∗ in the cell medium. Moreover, in contrast to the cell lysate blots, we did not detect any recombinant NTF control spanning from the +1 position of the predicted GPS to the N-terminus in the cell medium samples (Figures 1D and 1E).

Together, our immunoblot data show that GPR125 is cleaved near its GPS and that the majority of the ncNTF is shed *in vitro*.

### GPR125 peptides are found in human cerebrospinal fluid

GPR125 is highly expressed in the epithelial cells of the choroid plexus.^4^ Based on our data, several options exist for GPR125 expression^1^: it could be expressed as a full-length receptor,^2^ a complex of cleaved NTF:CTF, or^3^ an independent membrane ncCTF and a released ncNTF form. We used HIBCPP cells, derived from a human choroid plexus papilloma, and antibodies targeting the GPR125 ncNTF and ncCTF to understand which forms exist endogenously.^50^ Immunofluorescent staining of permeabilized HIBCPP cells detected the ncCTF but not the ncNTF, suggesting that GPR125 is cleaved and that the ncNTF is shed (Figure S1A).

We validated the specificity of the anti-GPR125-ncNTF antibody in HEK293T cells transiently expressing a fluorescently tagged hGPR125 with an N-terminal mCherry- and a C-terminal eGFP-tag (Figures S1B and S1C). First, the colocalization of the mCherry- and eGFP-tagged construct confirmed that GPR125 is incompletely cleaved and shed in the transient expression system, as we were able to detect GPR125 FL or a complex of ncNTF with ncCTF (Figures S1B and S1C). Moreover, orthogonal views of the HIBCPP cells showed that the GPR125 ncCTF was detected throughout the cell, including at the apical side (Figure S1A), which abuts the cerebrospinal fluid in physiological conditions (Figure 1F). We, therefore, investigated whether the ncNTF could be identified in human cerebrospinal fluid by MS analysis of samples from 131 healthy individuals. Indeed, we identified four different tryptic GPR125-derived peptides, of which three tryptic peptides belonged to the receptor ncNTF (Figures 1G, S2A, and S2B). In 24% of our samples, low levels of one C-terminal tryptic peptide were detected, while no C-terminal tryptic peptides were found in 76% of samples. In contrast, a smaller subset of samples (5–18%) contained substantially higher levels of three different N-terminal peptides.

### Cell medium containing the N-terminal fragment of GPR125 promotes cell survival of serum-starved cells

We next asked whether GPR125 ncNTF could regulate cell behavior beyond its site of expression. Shed GPR125 ncNTF was harvested from HEK293 cells expressing GPR125 and added to separate cell cultures that either expressed GPR125 FL, GPR125 CTF, or a negative control (mock transfectants). The added ncNTF consistently improved cell viability, even when added to cells already producing it via GPR125 FL expression (Figure 1H).

### The N- and C-terminal fragments of human GPR125 are found separately inside the cell

To explore the localization of the different receptor fragments, we analyzed double fluorophore-tagged GPR125-expressing HEK293T cells using confocal microscopy. Without cell permeabilization, we detected distinct cellular localizations of the GPR125 forms. The GFP-tagged ncCTF was mainly present in the perinuclear region, likely the ER-Golgi network, while the mCherry-tagged ncNTF was mostly seen in intracellular vesicles (Figure S3A and S3B). With permeabilization using Triton X-100, ncNTF was no longer detected in vesicles, suggesting that the soluble ncNTF was released during the permeabilization. This is striking since permeabilization has commonly been used in such investigations^19^^,^^38^; our results suggest this step obscures the actual cellular locations of GPR125 fragments.

Using western blot analysis of cell lysates and supernatants from cells expressing the double fluorophore-tagged GPR125 construct, we confirmed that GPR125 was cleaved. Immunoblot analysis also showed that the mCherry fluorophore was not cleaved from the receptor, given the absence of an mCherry band at ∼27 kDa (Figure S3C).

To avoid permeabilization while investigating the cellular distribution and internalization of the GPR125 fragments using live cell microscopy, we applied two extracellular tags: a FLAG tag (labeling the ncNTF) and an α-bungarotoxin binding site, as described in^30^ in extracellular loop 2 of GPR125 (labeling the ncCTF) (Figure 2A). This design allows staining of the different GPR125 forms expressed at the plasma membrane without cell permeabilization, enabling tracking of their movement from the plasma membrane over time.

**Figure 2:**
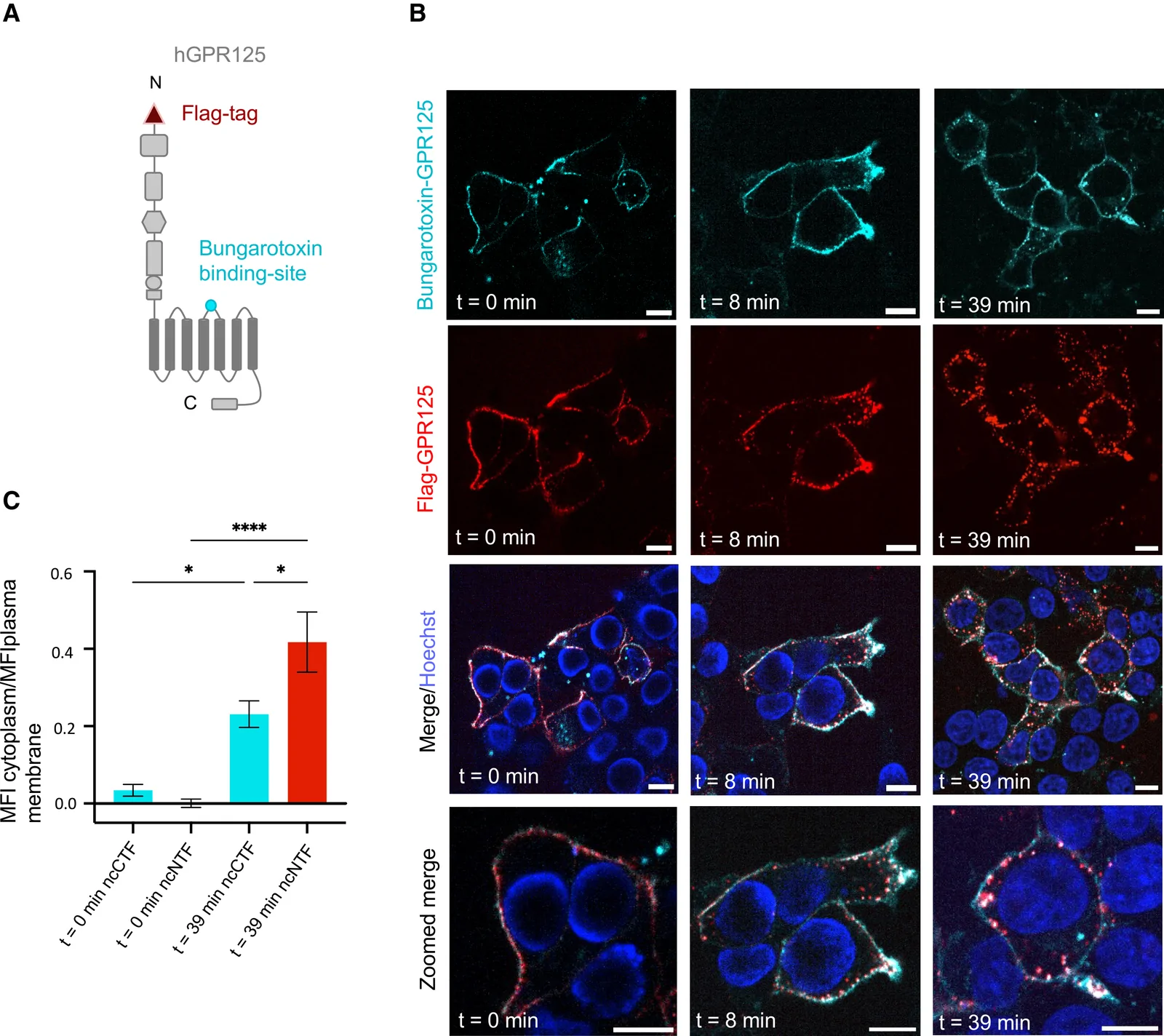
The human GPR125 ncNTF and ncCTF move separately within the cell (A) Schematic overview of hGPR125 with an N-terminal FLAG tag and an α-bungarotoxin binding site in extracellular loop 2 (ECL2). (B) hGPR125 ncNTF and ncCTF can separate and move independently within the cell. Live-cell microscopy of HEK293T cells expressing hGPR125 is shown in (A). The anti-Flag detects the N-terminal tag of GPR125, and the α-bungarotoxin detects the binding site in ECL2. Hoechst was added for nuclear counterstain. The width of the scale bar is 10 μm. (C) The ncCTF and ncNTF internalize over time. Quantification of the mean fluorescent intensity (MFI) of the fluorophores at the plasma membrane and in the cytoplasm at time points 0 min and 39 min. Data are presented as mean ± SEM of *n* = 10 quantified cells; ∗*p* < 0.05, ∗∗∗∗*p* < 0.0001, by one-way ANOVA followed by Tukey’s multiple-comparisons test. Non-significant *p* values are not shown. Full statistical details are provided in Table S4. See also Videos S1, S2, S3, S4, S5, and S6 and Figure S3.

Immediately after staining at 4°C (0 min), we observed an overlap of the Flag and the α-bungarotoxin labels, indicating expression of an FL GPR125 or non-covalently associated ncNTF/ncCTF in the plasma membrane (Figure 2B, left column). Next, we increased the temperature in the microscope heating chamber to 37°C to allow internalization. Within a few minutes, the fluorophores internalized and moved gradually apart, indicating a separation of the ncNTF and ncCTF (Figure 2B, middle column). After 39 min, the ncNTF was mainly located in intracellular vesicles or present in the cell medium (Figure 2B, right column; Videos S1, S2, S3, S4, S5, and S6). The ncCTF co-localized with the ncNTF in some intracellular vesicles but was mainly localized in the plasma membrane. Comparison of mean fluorescent intensity inside the cytoplasm versus the plasma membrane confirmed this observation (Figure 2C). We validated by immunoblotting that the construct used for live-cell microscopy was cleaved and that the ncNTF was shed into the cell medium (Figure S4). Our observation of a separation between ncNTF and ncCTF on plasma membranes during direct cell-cell contact, as well as in cells without direct cell-cell contact, suggests that ligand engagement or mechanical force in *trans* is not necessary for this separation (Figure 2B).

Video S1. Live-cell microscopy shows extracellular loop 2 labeling of hGPR125 in HEK293T cells, related to Figure 2

Video S2. Live-cell microscopy shows N-terminal FLAG labeling of hGPR125 in HEK293T cells, related to Figure 2

Video S3. Live-cell microscopy shows merged extracellular loop 2, N-terminal FLAG, and nuclear labeling of hGPR125 in HEK293T cells, related to Figure 2

Video S4. Live-cell microscopy shows extracellular loop 2 labeling of hGPR125 in HEK293T cells at lower acquisition speed, related to Figure 2

Video S5. Live-cell microscopy shows N-terminal FLAG labeling of hGPR125 in HEK293T cells at lower acquisition speed, related to Figure 2

Video S6. Live-cell microscopy shows merged extracellular loop 2, N-terminal FLAG, and nuclear labeling of hGPR125 in HEK293T cells at lower acquisition speed, related to Figure 2

Taken together, both microscopy approaches show that ncNTF and ncCTF are found separately within the cell.

### GPR125 is not cleaved at the predicted GPS

Mutations in the GPS have been reported to abolish cleavage of several aGPCRs, including GPR125.^19^^,^^22^^,^^51^^,^^52^^,^^53^^,^^54^ However, the N-terminal fragments were often measured only in cell lysates, not in the medium. Consequently, it is possible that the observed reduction of N-terminal fragment concentrations in the GPS-mutated cell lysates reflected altered shedding, rather than impairment of cleavage. We therefore investigated whether mutations in the GPS of GPR125 affected the amount of shed ncNTF. To this end, we designed 17 GPS mutants of the wt SLS triad with substitutions that altered hydrophobicity, length, and bulkiness of distinct residues.

Initially, we used *Xenopus laevis* oocytes as they can produce high, transient levels of GPR125 expression, enabling a high signal-to-noise ratio in western blot analysis. We identified three mutants (SLH, SLP, and SLR) that exhibited a significant decrease in ncNTF levels in cell lysates, while ncCTF levels remained unchanged (Figures S5A and S5B). This suggests that cleavage was unaffected, but shedding was indeed elevated.

We next investigated the GPR125 mutants in HEK293T cells using the same mutations, and two additional ones: the HLT mutant, representing the typical, cleavable GPS amino acid sequence of many aGPCRs; and the AAA mutant, which was previously described as abolishing cleavage in GPR125.^19^ Immunoblotting showed that all five GPR125 mutants were cleaved, as we were able to detect ncCTF and ncCTF∗ bands (Figure 3A). For one mutant, SLP, we noticed an ncCTF∗ band with an apparently slightly higher mass (Figures 3A and S5B). However, this mass shift disappeared after PNGase F treatment of HEK293T cell lysates, suggesting that the GPS may also affect the glycosylation pattern of the ncCTF∗ (Figure S5C).

**Figure 3:**
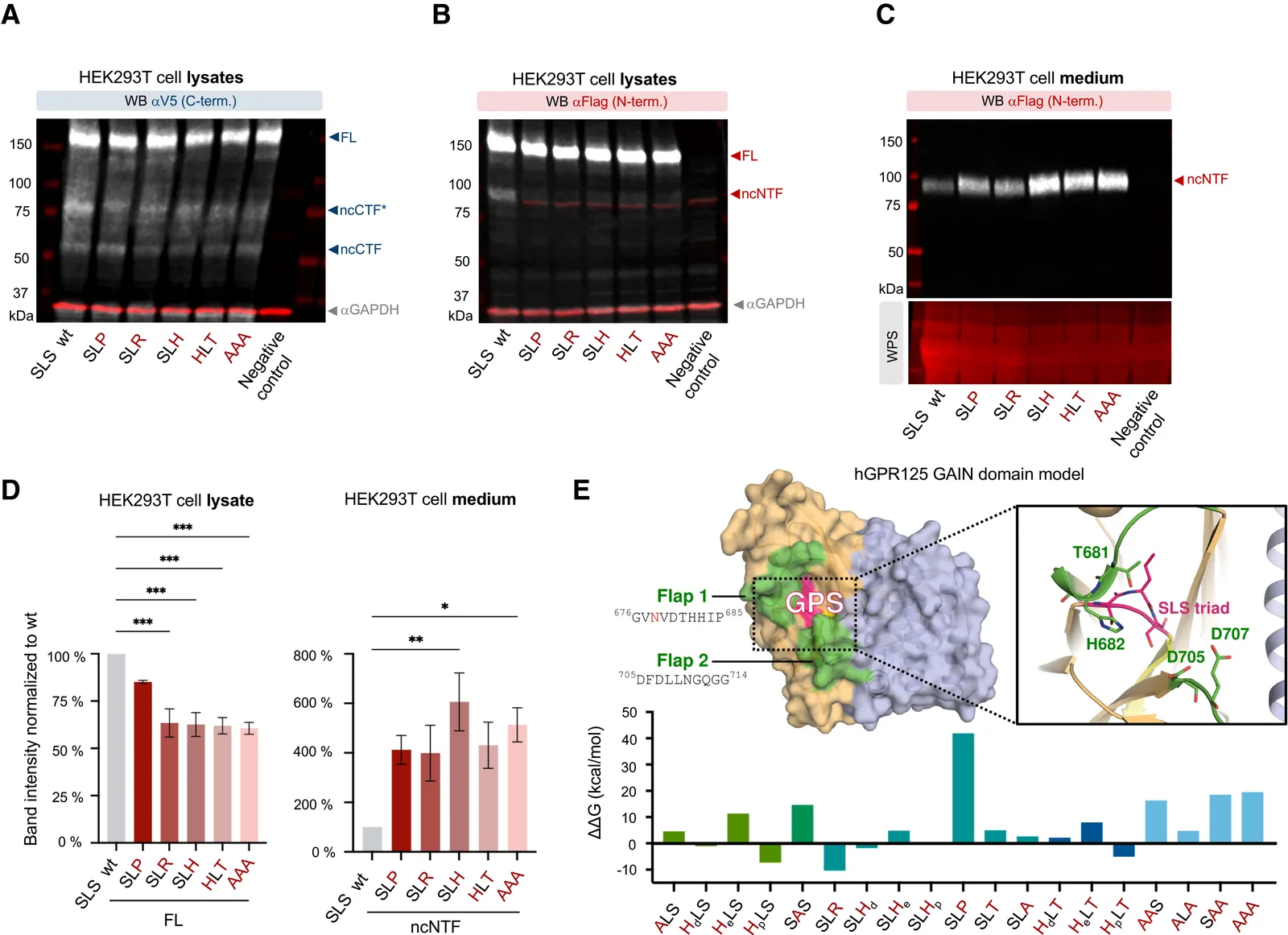
GPR125 is not cleaved at the GPCR autoproteolysis site (A–D) Mutations in the GPS of hGPR125 do not inhibit cleavage but lead to enhanced release of the cleaved ncNTF. (A) Immunoblot analysis of HEK293T cell lysates expressing hGPR125 wt and GPS mutants. The anti-V5 detects the C-terminal tag of hGPR125. The negative control shows cell lysates of cells expressing the empty vector (pcDNA3.1+). Anti-GAPDH has been included as a loading control. Arrows indicate the FL, ncCTF, and ncCTF∗. (B) Immunoblot analysis of HEK293T cell lysates with Flag-tagged proteins. The anti-Flag detects the N-terminal tag of hGPR125. The negative control shows cell lysates from cells expressing the empty vector (pcDNA3.1+). Anti-GAPDH has been included as a loading control. Arrows indicate the FL and ncNTF. (C) Immunoblot analysis of supernatant from HEK293T cells expressing hGPR125 wt and GPS mutants. The anti-Flag detects the N-terminal tag of hGPR125. The negative control shows cell lysates of cells expressing the empty vector (pcDNA3.1+). A whole-protein stain (WPS) was performed as a loading control. Arrows indicate the hGPR125 ncNTF. (D) Quantification of B (left) and C (right). Data are presented as mean ± SEM of *n* = 3 independent experiments; ∗*p* < 0.05, ∗∗*p* < 0.01, and ∗∗∗*p* < 0.001 by one-way ANOVA followed by Dunnett’s multiple-comparison test compared to the wt. Non-significant *p* values are not shown. Full statistical details are provided in Table S4. (E) hGPR125 GAIN domain stability is affected by mutations in the GPS. GAIN domain homology model showing the predicted effects of GPS mutations on protein stability measured as the difference in the PRIME energy function and corrected for protein solvent-accessible surface area.^55^^,^^56^^,^^57^ A higher ΔG than for the unmutated model, indicative of lower overall stability, is observed for all mutations within the GPS triad except HLS and SLR. Histidine mutations are shown by protonation state: H_p_ (both nitrogens protonated), H_e_ (proton at Nε), and H_d_ (proton at Nδ). See also Figure S4 and Table S1.

Consistent with the data from expression in oocytes, the amount of shed ncNTF in the cell medium was greater for all mutants compared to the wt and significantly increased for the SLH and AAA mutants (Figure 3C). Furthermore, we were barely able to detect any ncNTFs of the wt or mutants in the cell lysates (Figure 3B).

These results suggest that GPR125 is cleaved by a mechanism distinct from the previously reported GPS-dependent autoproteolytic cleavage, and that its GPS does not control the cleavage event as such. However, the level of shed ncNTF is affected by mutations in the GPS, implying that this motif is important for the interaction between the ncNTF and the ncCTF.

### Alterations in the GPS site of GPR125 affect protein stability

The GPS is located in a mostly enclosed space within the NTF and is only exposed on the NTF surface within a groove enclosed by two flexible loop regions called Flap1 and Flap2 in the GAIN domain.^30^ Studies show that the β-sandwich fold of the GAIN domain tightly encloses the Stachel sequence at its most C-terminal β-strand via a plethora of hydrophobic interactions, thus forming a non-covalent NTF:CTF complex.^18^^,^^44^^,^^58^^,^^59^

One possible explanation for increased ncNTF shedding induced by GPS mutations is that these interfere with the ncNTF:ncCTF interface. We therefore modeled the structures of GPR125 GAIN domain mutants, hypothesizing that mutations predicted to affect stability or solvent accessibility would alter local stability by inducing differences in Gibbs free energy (ΔΔG) (Figure 3C).

We computationally approximated the impact of mutations on the GPR125 GAIN domain by introducing them into a structural model and integrating relative Gibbs free-energy values from various polar, nonpolar, and solvent interactions to estimate local differences in stability. The data confirm that all mutations within the GPS triad, except HLS and SLR, destabilize the protein, implying easier ncNTF separation from a destabilized ncNTF/ncCTF complex (Figure 3E; Table S1).

Unexpectedly, the SLR mutation led to a more stable local environment, mainly due to a decrease in solvation energy and a higher contribution of van der Waals interactions relative to the wt (Table S1). We predicted that the AAA mutation would destabilize interactions of the protein with the lipophilic environment, likely due to disruption of lipid interactions with the bulky hydrophobic leucine side chain. Mutations of the serine at the third position, directly C-terminal to the expected cleavage site, had little overall effect on stability except for the SLP mutation, for which major destabilizing effects were predicted. Looking at individual stability contributions, the SLH mutations had strong impacts on covalent stability (between +14.1 and +19.4 kcal/mol); however, solvation effects mitigated differences in protein stability (between −1.8 and +4.9 kcal/mol; Table S1).

The SLR, SLP, and SLH mutations all replace the small serine side chain with a bulky one, and the proline further disrupts the protein backbone orientation. Mutation of the wt non-conventional GPS triad to the conserved GPS triad by mutating it to HLT, HLS, or SLH showed an overall low impact on predicted stability, whereas the protonation state of the histidine had an impact on stability; HSP (both nitrogens protonated) stabilized, and HSE (proton at Nε) destabilized, while HSD (proton at Nδ) had a very low overall impact on stability (Table S1).

Taken together, these data support the hypothesis that mutations in the GPS can affect the protein’s overall stability.

### The extracellular region regulates GPR125 cleavage and ncNTF release

To further investigate the impact of the GPS on shedding, we compared cleavage and shedding patterns of human, mouse, rat, and zebrafish GPR125. The extracellular domains of the mammalian sequences exhibit high similarity (Figure 4A). However, whereas the GPS of the human receptor is SLS, it is SLG for the rat and mouse and SLN for the zebrafish GPR125. Upon expression in HEK293T cells, we found that human and mouse GPR125 shed their ncNTF into the cell medium, whereas the rat did not (Figure 4B). Analysis of the corresponding cell lysates showed that the human and mouse receptors were robustly cleaved, while rat GPR125 was only cleaved to a very small extent (Figures S6A and S6B). The zebrafish GPR125 shares a low sequence similarity with the mammalian GPR125 and displayed differences in cleavage and shedding patterns, suggesting that it is not a suitable model to understand human GPR125 processing (Figures 4A, 4B, S6A, and S6B).

**Figure 4:**
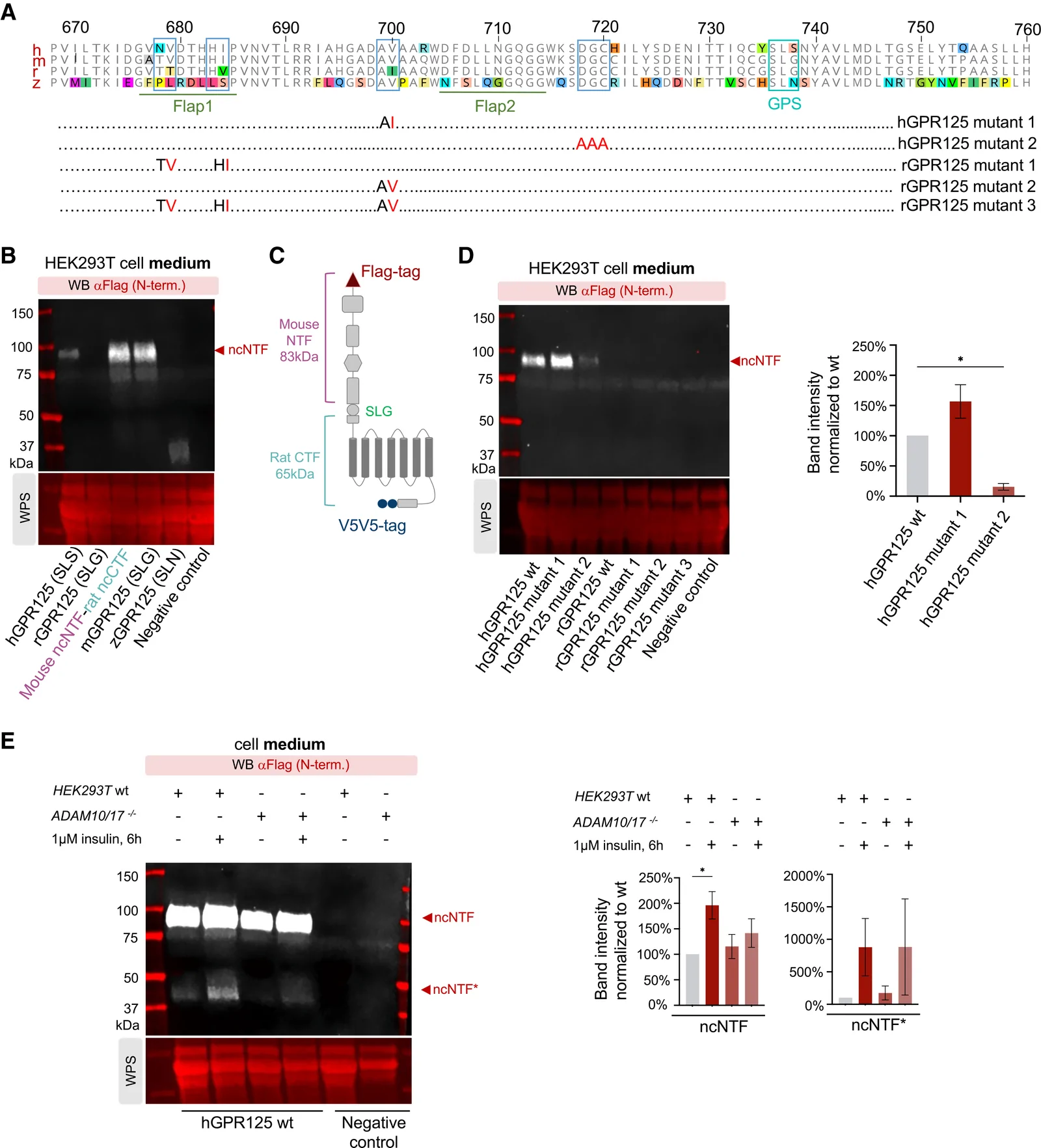
The extracellular region regulates GPR125 cleavage and ncNTF release (A) Sequence alignment of the extracellular region (ECR) of the human (h), mouse (m), rat (r), and zebrafish (z) GPR125. The alignment shows the most C-terminal part of the ECR and includes Flap1, Flap2, and GPS. The blue boxes indicate the sites of mutagenesis shown in D. The colored amino acids indicate the disagreements from the consensus sequence. (B and C) The ncNTF controls cleavage and shedding of GPR125. Immunoblot comparing the amount of shed GPR125 ncNTF for the different species and the chimeric receptor shown in C. The anti-Flag antibody detects the N-terminal tag of the GPR125 receptors. The negative control shows cell lysates of cells expressing the empty vector (pcDNA3.1+). A whole-protein stain (WPS) was performed as a loading control. Arrow indicates the shed ncNTFs. (D) Mutations in the ECR of hGPR125 can affect the amount of shed ncNTF. Immunoblot comparing the amount of shed human and mouse GPR125 ncNTF for different mutations in the ECR. The anti-Flag antibody detects the N-terminal tag of the GPR125 receptors. The negative control shows cell lysates of cells expressing the empty vector (pcDNA3.1+). A whole protein stain (WPS) was performed as a loading control. The arrow indicates the shed ncNTF. Data are presented as mean ± SEM of *n* = 3 independent experiments; ∗*p* < 0.05 by one-way ANOVA followed by Dunnett’s multiple-comparisons test compared to the wt. Non-significant values are not shown. Full statistical details are provided in Table S4. (E) Insulin increases the amount of shed ncNTF and ncNTF∗. Immunoblot showing the amount of shed hGPR125 ncNTF and ncNTF∗ in HEK293T *wt* cells and *ADAM10/17*^−/−^ cells. The anti-Flag antibody detects the N-terminal tag of the hGPR125 receptors. The negative control shows cell supernatant from cells expressing the empty vector (pcDNA3.1+). A whole-protein stain (WPS) was performed as a loading control. The arrows indicate the shed ncNTF and ncNTF∗. Data are presented as mean ± SEM of *n* = 5 (ncNTF) or *n* = 3 (ncNTF∗); *p* < 0.05 by one-way ANOVA followed by Dunnett’s multiple-comparisons test compared to the wt. Non-significant *p* values are not shown. Full statistical details are provided in Table S4. See also Figures S5 and S6.

Despite sharing the same GPS, rat and mouse GPR125 differ in cleavage patterns (Figure 4C). To investigate the contribution of receptor domains to shedding and cleavage events, we replaced rat ncNTF with the mouse ncNTF in rat GPR125. This chimeric GPR125 construct (mouse ncNTF-rat ncCTF) was cleaved, and its ncNTF was shed (Figures 4B, C, S6A, and S6B), indicating that the ncNTF controls cleavage in a GPS-independent manner.

Since we detected two bands corresponding to the GPR125 FL of the mouse, rat, and zebrafish receptors, we treated cell lysates with PNGase F to remove oligosaccharides before immunoblotting to determine whether these bands represent alternative glycosylation forms versus cleavage forms (Figures S6A and S6B). We detected a large shift in the ncCTF∗ of mouse GPR125 after treatment, but no apparent shift in the ncCTF, suggesting that, such as human GPR125, these bands represent separate cleavage products (Figure S6C). The additional GPR125 FL band we observed for the mouse and rat disappeared after treatment, indicating that the FL exists in two distinct glycosylation forms in these species.

Together, these results indicate that the ncNTF controls GPR125 cleavage in a GPS-independent manner.

### Distant regions of the ncNTF regulate the cleavage of GPR125

We next aimed to understand why relatively minor amino acid differences in the ncNTF of rat, rGPR125, and mouse, mGPR125, receptor homologs led to differential cleavage patterns. We identified a possible explanation within the Flap1 region using NetOGlyc-4.0,^60^ where the rat sequence GVTTDT is a better predicted candidate for O-glycosylation than GVTVDT in mice and GVNVDT in humans (Figures 4A and S6D). Glycosylation of Flap1 could conceivably control access of solvent and other enzymes to the region around the GPS.

To test this hypothesis, we mutated the Flap1 motif of the rat receptor to be either more similar or identical to the human or mouse Flap1 motif. We also identified a two-amino acid motif located between the GPS and the Flap1 region (positions 699–700), which is identical (AV) in the cleavable human and mouse GPR125 but different (AI) in the uncleavable rat GPR125, and therefore potentially involved in the cleavage of GPR125. We mutated the rGPR125 motif AI to AV, and the AV in hGPR125 to the rat receptor’s AI. Moreover, we mutated the triple amino acid motif DGC (positions 718–720) of the human receptor to AAA, as this triad has previously been described to be autoproteolytically cleaved in a different protein.^61^

For rGPR125, none of these mutations affected the amount of ncCTF or ncCTF∗, nor did they lead to a detectable, shed ncNTF. For hGPR125, these mutations did not significantly affect the amount of ncCTF or ncCTF∗. The amount of shed ncNTF was, however, significantly decreased for the hGPR125 AAA mutant. (Figures 4D, S6E, and S6F).

These results indicate that motifs in the extracellular region rather than single residues (such as in the GPS triad), control GPR125 cleavage.

### A role of cellular proteases and insulin in the shedding of the human GPR125 ncNTF

There is a growing body of evidence for the release of extracellular fragments of aGPCRs by proteases.^26^^,^^27^^,^^47^ Since our results suggest that GPR125 is not cleaved by the previously described autoproteolytic process at the GPS,^19^ we tested the impact of several different metalloprotease and aspartate protease inhibitors on GPR125 cleavage and ncNTF release. In most experiments, a broad-spectrum metalloprotease inhibitor, an ADAM10 inhibitor, and a general beta-secretase (BACE) inhibitor only moderately reduced the level of ncNTF in the culture medium. However, when GPR125-expressing HEK293T cells were cultured in the presence of an inhibitor of both ADAM17 and ADAM10, a strong decrease in the level of ncNTF in the culture medium was always observed, suggesting the involvement of ADAM17 in the shedding of the ncNTF (Figure S7A). Although the inhibition of ADAM17 hampered the release of the ncNTF, the levels of the ncCTF and ncCTF∗ in the cell lysate were not significantly affected. In contrast, inhibition of BACE modestly reduced the level of ncNTF in the culture medium and substantially downregulated the levels of ncCTF, but not ncCTF∗, in the cell lysates (Figures S7A and S7B).

As the combined inhibition of ADAM17 and ADAM10 had the most pronounced effect on GPR125 ncNTF shedding, we employed an ADAM10/17-deficient HEK293T cell line^62^ to further investigate the involvement of these proteins. We used the anti-aging protein Klotho, which is also expressed in the epithelial cells of the choroid plexus,^63^^,^^64^ as a positive control for ADAM10/17-mediated cleavage. Since insulin has previously been shown to increase the amount of Klotho shed in an ADAM10/17-dependent manner,^64^ and to be secreted from the epithelial cells of the choroid plexus,^65^ we included it as a possible modulating factor for GPR125.

We expressed GPR125 and the membrane-anchored form of Klotho in HEK293T *wt* cells and ADAM10/17^−/−^ cells. As expected, Klotho was cleaved and shed from HEK293T *wt* cells but not in the ADAM10/17^−/−^ cells (Figures S7C and S7D). However, GPR125 still shed its ncNTF in ADAM10/17^−/−^ cells (Figure 4E), indicating that ADAM10/17 alone might not be responsible for ncNTF shedding.

The addition of insulin significantly increased the amount of shed ncNTF in the cell medium of HEK293T wt cells, but not in ADAM10/17^−/−^ cells, whereas the levels of FL, ncCTF, and ncCTF∗ remained unaffected (Figures 4E, S7E, and S7F). In some experiments, we also noted that insulin incubation led to a clearly detectable GPR125 ncNTF∗ fragment of around 45 kDa (Figure 4E). The ncNTF∗ band differed from the theoretical size of the ncNTF∗ band (68 kDa), which would match the ncCTF∗ (see Figures 1A and 1B), suggesting that ncNTF∗ might be cleaved by additional proteases.

Together, these results suggest that cellular proteases contribute to the shedding of extracellular GPR125 fragments. Moreover, insulin regulates the amount of soluble ncNTF.

### GPR125 has at least one additional cleavage site

To identify the cleavage sites of GPR125, medium from native or GPR125-expressing HEK293 cells was analyzed by LC-MS/MS after protein digestion using either trypsin or TrypN, followed by enrichment for N/C-terminal peptides. These analyses led to the identification of peptides primarily originating from the ncNTF corresponding to the immunoblot staining. Multiple endogenous cleavage fragments from possible cleavage sites were detected, with one site identified by both the neo-N-terminal and neo-C-terminal peptides at position 343–344 in the sequence DIVVLE|SSACYQ (Figures 5A–5D; Table S2). The hypothetical size of a cleavage fragment spanning the sequence between the N-terminus and amino acid 344 is ∼34 kDa, consistent with the observed size of the ncNTF∗, considering the possibility of post-translational modifications, including glycosylation. As there are no predicted trypsin and TrypN-cleavage sites in close proximity to the GPS (Figure S8), additional LC-MS/MS analyses were carried out using GluC as the digestive enzyme. However, this resulted in the detection of only a few peptides, none of which covered the region around the GPS. Consequently, these experiments did not allow identification of the exact position of the second cleavage site closely located to the GPS (Table S3).

**Figure 5:**
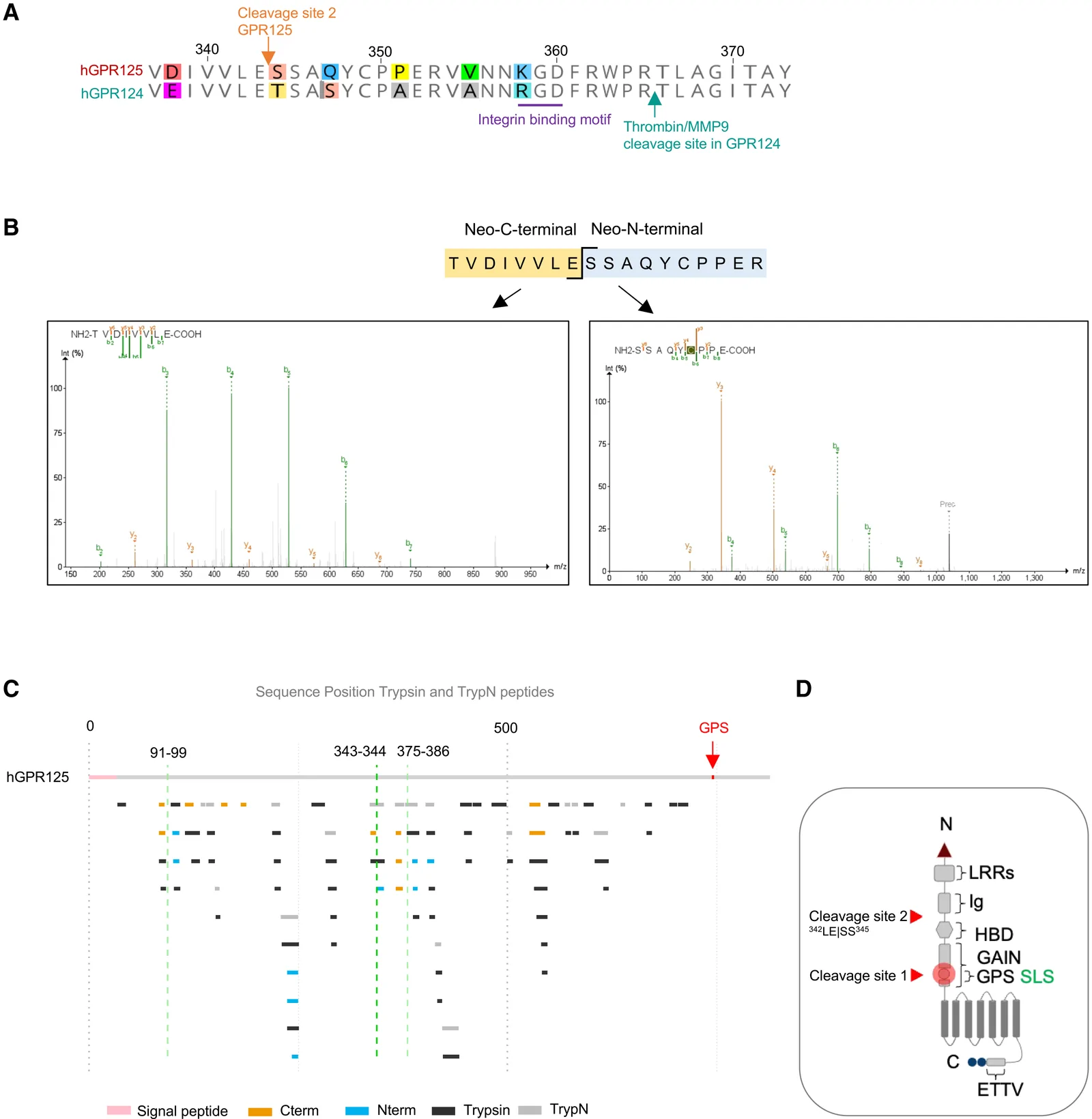
GPR125 has a second non-conventional cleavage site (A) Sequence alignment of hGPR125 and hGPR124 indicating the exact location of the newly identified second cleavage site and the previously identified cleavage site in hGPR124. The colored amino acids indicate differences between the two sequences. hGPR125 has a suspected alternative integrin-binding motif at the position where hGPR124 has been shown to have a regular integrin-binding motif. (B) MS/MS spectra of the neo-N-terminal and neo-C-terminal peptides supporting identification of the newly identified cleavage site 2 obtained through digestion with either trypsin (left) or TrypN (right). The two identified peptides show complete ion coverage across the site of cleavage and were identified in at least 2/3 individually grown hGPR125-expressing HEK293 cell medium samples. (C) Overview of GPR125 peptides identified by LC-MS/MS from hGPR125-expressing HEK293 cell medium digested with either TrypN or Trypsin. The green dashes represent sites where a neo-C-terminus (Cterm) and a neo-N-terminus (Nterm) have been identified in close proximity along the protein sequence. Blue colored lines show neo-N-terminal peptides, orange lines show neo-C-terminal peptides, black lines show internal peptides originating from digestion with trypsin, and gray lines show internal peptides originating from digestion with TrypN. (D) Schematic overview of hGPR125 indicates the exact location of the cleavage site 2 and the approximate location of the cleavage site 1 in hGPR125. See also Tables S2 and S3.

## Discussion

Our study demonstrates that (1) GPR125 is cleaved in at least two places but not at the GPS. (2) Once cleaved, the ncNTF and ncCTF can localize separately within the cell, and shedding occurs. The GPS impacts, but is not required, for ncNTF release. (3) Insulin either stimulates shedding or plays a role in preserving ncNTF after shedding, and (4) shed ncNTF can promote cell survival of serum-starved cells *in vitro* and may play a role *in vivo*, as (5) we identified GPR125-derived peptides in human cerebrospinal fluid.

Our data indicate that GPR125 is not autoproteolytically cleaved, unlike conventional aGPCRs. A previous study concluded the opposite—that GPR125 undergoes autoproteolytic cleavage at its GPS—on the basis that mutations at this site diminished the level of ncNTF found in cell lysate^19^; however, the cell medium was not examined in that study. In addition to showing that GPS mutants of GPR125 are still cleaved to a similar extent as the wt receptor, our analysis revealed that some mutants modulate the amount of ncNTF in the cell medium, suggesting that the GPR125 GPS regulates shedding rather than acting as a cleavage point or regulating cleavage. Furthermore, mutating the GPR125 GPS (SLS) to match the conventional motif (HLT) did not affect cleavage but did increase the amount of shed ncNTF. Structural modeling suggests that the GPS, along with other GAIN domain regions between the GPS and Flap1, is involved in stabilizing the ncNTF-ncCTF complex.

If conventional autoproteolysis is not the mechanism behind GPR125 cleavage, proteases may be involved. This has been seen before in adhesion family members.^24^^,^^26^^,^^27^^,^^47^ Although we could not definitively identify proteases responsible for GPR125 cleavage, our data implicate ADAM17, possibly in conjunction with other proteases. MS analysis of GPR125 in HEK293 cells revealed free N- and C-terminal forms at several positions N-terminal to the GPS, which could indicate the involvement of multiple proteases in GPR125 cleavage.

The separate localization of the GPR125 ncCTF and ncNTF is in agreement with previous reports on the neighboring ADGRL subgroup members, where the fragments behave as separate proteins in cell-surface interactions, signaling, and internalization.^66^ ADGRE2/EMR2 provides another example of distinct cellular localization for the NTF and CTF, each exhibiting independent distribution within lipid raft microdomains.^67^

In the cell, GPR125 ncNTF mainly localized in intracellular vesicles. This adds a layer of intrigue, especially when considered alongside our finding that GPR125 ncNTF release is increased in the presence of insulin. An increase is also seen in the Klotho protein, included as a positive control in our experiments. While the mechanism is not well understood, insulin may induce the movement of vesicle-associated proteins to the cell surface by enhancing the endocytic recycling pathway, much as it facilitates glucose uptake by stimulating translocation of GLUT4-containing vesicles to the plasma membrane.^68^ Indeed, there is already evidence that this effect on endocytic recycling extends to cell types that do not express GLUT4.^69^^,^^70^ In addition to its best-known role in energy regulation, insulin reduces brain inflammation and improves recovery after traumatic brain injury.^71^ Furthermore, it is released from the choroid plexus in response to serotonergic signaling.^65^

The choroid plexus lines the ventricles of the brain, producing cerebrospinal fluid and serving as a crucial source of bioactive molecules for brain development, function, and repair. High GPR125 expression here hinted that ncNTF might be shed into the cerebrospinal fluid. Confirming this, we identified three tryptic peptide fragments arising from the N-terminal fragment of GPR125 in human cerebrospinal fluid. At much lower sensitivity, we also identified a tryptic fragment arising from the intracellular region, presumably arising from cells and/or membrane fragments in the cerebrospinal fluid. However, further experiments would be needed to demonstrate that the ncNTF is shed into the cerebrospinal fluid with biological significance. We learned that shed GPR125 ncNTF can carry out a biological function independently of the ncCTF, represented by a positive effect on cell viability, and since the NTF/ncNTFs of several other aGPCRs have been shown to have distinct biological functions, we hypothesize that the soluble GPR125 ncNTF could have a role defined by its unique domains,^72^ perhaps facilitated by cerebrospinal fluid transport.

The biological functions of aGPCR NTF/ncNTFs range from control of the CNS neuron pool size^39^ to critical participation in heart development independent of the CTF.^73^ Vasculostatin, the soluble NTF of the ADGRB1/BAI1, inhibits endothelial cell migration *in vitro*, reduces angiogenesis *in vivo,* and inhibits intracranial glioma growth,^48^^,^^49^ showing therapeutic potential for cancer therapy.^74^ Perhaps the strongest clue to the function of GPR125 ncNTF comes from its nearest neighbor, GPR124, whose soluble ncNTF is released by endothelial cells during capillary-like network formation and in response to growth factor stimulation. The GPR124 ncNTF binds glycosaminoglycans, and further processing with matrix metalloprotease 9 yields a form of the fragment that mediates endothelial cell survival by linking integrin *a*_*v*_*b*_*3*_ to glycosaminoglycans.^47^

To conclude, we find that cleavage does not require the GPS, but mutations in this site destabilize the interaction between the ncNTF and ncCTF. Proteases are involved in the release, which is further regulated by insulin. The shed GPR125 ncNTF can carry out a biological function independently of the ncCTF. These observations may reveal new therapeutic targets for diseases involving barriers and cerebrospinal fluid dynamics. They moreover highlight that a one-size-fits-all model likely does not explain the mechanisms behind aGPCR topologies, which instead appear unique to different receptors.

A key question remains as to the roles of the shed GPR125 ncNTF and the membrane-bound ncCTF. Given the wide variety of functions seen for soluble (nc)NTF forms of other aGPCRs, and the positive effect of the GPR125 ncNTF on *in vitro* cell viability or possibly cell proliferation, the GPR125 ncNTF may well serve an important biological purpose—for instance, in CNS development and function, given the presence of GPR125 peptides in human cerebrospinal fluid. Further investigation is warranted to fully explore these possibilities.

### Limitations of the study

One limitation of this study is that we were unable to identify the exact cleavage site responsible for the generation of the major ncCTF species proximal to the GPS region. Although our data strongly support cleavage independent of conventional GPS autoproteolysis, further studies are needed to fully characterize the cleavage mechanism. A second limitation is that the protease or proteases responsible for GPR125 processing remain unidentified. Inhibitor studies and experiments in ADAM10/17-deficient cells suggest that multiple proteolytic activities may contribute to cleavage and shedding. Furthermore, although insulin increased extracellular ncNTF levels, the underlying mechanism remains unclear. Finally, while we demonstrate that conditioned medium containing shed GPR125 ncNTF increases cell viability *in vitro* and identify GPR125-derived peptides in human cerebrospinal fluid, the physiological functions of shed ncNTF and its significance *in vivo* remain to be determined.

## Resource availability

### Lead contact

Further information and requests for resources and reagents should be directed to and will be fulfilled by the lead contact, Mette M. Rosenkilde rosenkilde@sund.ku.dk.

### Materials availability

All unique/stable reagents generated in this study are available from the lead contact with a completed materials transfer agreement.

### Data and code availability

• Data: Uncropped western blot and microscopy images have been deposited at Mendeley Data: https://doi.org/10.17632/5jz9cmkpgv.1 and are publicly available. The raw mass spectrometry data generated in this study and used for the identification of potential GPR125 cleavage sites have been deposited in the ProteomeXchange Consortium via the PRIDE partner repository^55^ with the dataset identifier: PXD083139 and are publicly available. Enroll-HD clinical data are shared with the research community through specified datasets. Access to data may be obtained through request, subject to approval by the Scientific Review Committee that weighs the scientific merit of the proposed project against the increased risk for participant identification. Any researcher employed by a recognized research organization can open an Enroll-HD access account to obtain data from Enroll-HD, subject to the researcher’s employer/institution signing the appropriate data use agreement(s). More detailed access information is available at https://enroll-hd.org/for-researchers/data-from-human-samples/and https://enroll-hd.org/for-researchers/access-data/. The specific dataset “HDClarity Proteomics Data for CSF – Mass Spec Data” can be requested here https://enroll-hd.org/for-researchers/access-data-biosamples/.
• Code: No custom code was generated for this study.
• Other: Any additional information required to reanalyze the data reported in this paper is available from the lead contact upon request.

## Acknowledgments

We thank Søren Petersen, Maibritt S. Baggesen and Adrian Dragan for excellent technical assistance. We thank Dr. Irina Pozdnyakova from the Proteomics Research Infrastructure, University of Copenhagen, for performing the MS analysis of the purified NTF, Assoc. Prof. Hiren Jitendra Joshi from the University of Copenhagen, Denmark for support in the glycosylation prediction, Dr. Anca Stoica for her help with the *Xenopus laevis* oocytes, Prof. Steen G. Hasselbach from the Danish Dementia Research Center at Copenhagen University Hospital for his help to acquire human cerebrospinal fluid, Gunnar Kleinau and Patrick Scheerer from the Charité, Berlin for their valuable input on a potentially interesting GPR125 mutagenesis site and Emily Beaman from the University of Copenhagen, Denmark for critical reading and fruitful manuscript discussion. The cerebrospinal fluid data used in this work were generously provided by the participants in the Enroll-HD study and made available by 10.13039/100005725CHDI Foundation, Inc. Enroll-HD is a global clinical research platform intended to accelerate progress toward therapeutics for Huntington’s disease; core datasets are collected annually from all research participants as part of a multi-center longitudinal observational study. Enroll-HD is sponsored by CHDI Foundation, Inc., a nonprofit biomedical research organization exclusively dedicated to developing therapeutics for Huntington’s disease. Enroll-HD would not be possible without the vital contribution of the research participants and their families. The collection of cerebrospinal fluid samples and data used in this work would not be possible without the vital contribution of the research participants and their families in the HD-Clarity and HD-CSF studies. HD-Clarity and HD-CSF are cerebrospinal fluid collection initiatives designed to facilitate therapeutic development for Huntington’s disease. These initiatives are led by Dr. Edward Wild and sponsored by 10.13039/501100000765University College London. HD-Clarity is funded by CHDI Foundation, Inc. The 10.13039/501100000265Medical Research Council UK (MR/M008592/1) funded HD-CSF. Grants: MMR was supported from the 10.13039/501100000781European Research Council: VIREX (Grant agreement 682549, Call ERC-2105-CoG and the 10.13039/501100003554Lundbeck Foundation -(R268-2017-409) 113978). SB and MMR were supported by the B-ACTIVE network, a Horizon-MSCA-2022-Doctoral Network (project no. 101120187). AHS was supported by the Absalon Foundation of May 1^st^ 1978, and Toyota-fonden Danmark, 10.13039/501100001659Deutsche Forschungsgemeinschaft, CRC 1423, project 421152132 (A06), and project 542631574 (LA2861/10-1 to TL), 10.13039/501100001659Deutsche Forschungsgemeinschaft (DFG) through CRC 1423, project no. 421152132 (project A06 and and project 542631574 (LA2861/10-1) to TL and projects C01 and Z04 to PWH. The National Science Center, (Weave-UNISONO; UMO-2023/05/Y/NZ1/00227) to RMK. FWO-Vlaanderen Junior and Senior Research Grants (G058421N, G025923N, G036423N) and a KU Leuven C1 grant (14/23/143) to PP and PEM. MJD is supported by the 10.13039/501100009708Novo Nordisk Foundation (NNF20SA0064214). NWT was supported by the 10.13039/501100025321Danish Cardiovascular Academy which is funded by the 10.13039/501100009708Novo Nordisk Foundation (NNF20SA0067242) and the 10.13039/100007405Danish Heart Foundation. NMIT acknowledges support from the 10.13039/501100009708Novo Nordisk Foundation through the 10.13039/501100024571Center for Protein Research (CPR) (NNF14CC0001), a Hallas-Møller Emerging Investigator grant (NNF17OC0031006) and an NNF Hallas-Møller Ascending Investigator grant (NNF23OC0081528). N.M.I.T. is a member of the Integrative Structural Biology Cluster (ISBUC) at the University of Copenhagen. NHS and FQL were supported by the 10.13039/100005725CHDI Foundation (record no. # A-18952).

## Author contributions

Conceptualization, A.L.S.W., O.L., K.S., and M.M.R..; methodology, A.L.S.W., O.L., F.S., I.R., N.W.T., R.M.K., J.B., G.M.H., C.K.G., T.T.B., N.M.I.T., A.H.S., N.M., T.L., P.P., P.W.H., K.S., M.J.D., B.E., and P.E.M.; investigation, A.L.S.W., C.S., F.S., R.M.K., J.B., I.R., O.L., N.W.T., A.K.D., S.M.B., F.L.Q., T.T.B., E.S., S.B., and E.G.; proteomic analysis of human C.S.F.. F.L.Q., and N.H.S.; writing – first draft, A.L.S.W., O.L., and M.M.R.; writing – review and editing; all co-authors; funding acquisition, P.E.M. and M.M.R.; resources, H.I. and H.S. (HIBCPP cells) C.G. and J.L. (ADAM10/17 deficient cells); supervision, T.L., P.E.M., and M.M.R.

## Declaration of interests

The authors declare no competing interests.

## STAR★Methods

### Key resources table

**Table:** 

REAGENT or RESOURCE	SOURCE	IDENTIFIER
**Antibodies**		

Mouse anti-V5	Invitrogen	#R96025; RRID: AB_159313
Mouse anti-FlagM1	Sigma-Aldrich	#F3040; RRID: AB_439712
Mouse anti-FlagM2	Sigma-Aldrich	#F3165; RRID: AB_259529
Chicken anti-GAPDH	Sigma-Aldrich	#AB2302; RRID: AB_10615768
Rabbit anti-GPR125 NTF	Atlas antibodies	#HPA008904; RRID: AB_1849258
Goat anti-GPR125 CTF	Novus	# NBP1-03561
Mouse anti-mCherry antibody	Sigma-Aldrich	#SAB2702291
Mouse anti-eGFP antibody	Invitrogen	#MA1-952; RRID: AB_889471
α-bungarotoxin, Alexa Fluor^TM^ 488 conjugate	Invitrogen	#B13422
Alexa fluor 568-conjugated Goat anti-mouse	Jackson ImmunoResearch	#115–575–146; RRID: AB_3095451
Alexa fluor 647-conjugated Goat anti-mouse	Jackson ImmunoResearch	RRID: AB_2338902
mouse anti-E-Cadherin	BD Biosciences	#610181; RRID: AB_397580
Donkey anti-rabbit-647	Jackson ImmunoResearch	#711–606–152; RRID: AB_2340625
Donkey anti-mouse-Cy3	Jackson ImmunoResearch	#715–166–151; RRID: AB_2340817
Alexa fluor 488-conjugated anti-goat	Invitrogen	#A-11055; RRID: AB_2534102
Goat IgG	R&D	#AB-108-C; RRID: AB_354267
Rabbit IgG	R&D	#AB-105-C; RRID: AB_354266
IRDye 800CW Goat anti-Rabbit	Li-COR	#926–32211; RRID: AB_621843
IRDye 800CW Goat anti-Mouse	Li-COR	#926–32210; RRID: AB_621842
IRDye 680CW Donkey anti-Chicken	Li-COR	#9925–68075

**Biological samples**		

Human cerebrospinal fluid	HD-Clarity/HD-CSF (Enroll-HD)	NCT02855476

**Chemicals, peptides, and recombinant proteins**		

Tetracycline	ThermoFisher Scientific	#J61714
Hygromycin B	ThermoFisher Scientific	#10687010
Blasticidin	vwr	#APOSBIB4432
Insulin	Lonza	#BE02-033E20
Trypsin	Promega	#V5111
Glu-C	Promega	#V1651
TrypN	ProtiFi	#K001

**Critical commercial assays**		

Trans-Blot Turbo RTA Transfer Kit, LF PVDF	BIO RAD	#1704275
Rapid Gold BCA Protein Assay Kit	Pierce	#A53225, #A55860
mMESSAGE mMACHINE T7	Invitrogen	#AM1344
MEGAclear Transcription Clean-Up Kit	Invitrogen	#AM1908
ProteoExtract All-in-one Trypsin digestion kit	Calbiochem	#650212

**Deposited data**		

Uncropped Western blots and microscopy images	This paper	Mendeley Data, https://doi.org/10.17632/5jz9cmkpgv.1
Raw mass spectrometry data generated in this study used for the identification of potential GPR125 cleavage sites	This paper	Proteome Xchange via PRIDE, PXD083139

**Experimental models: Cell lines**		

Human: HEK293T cells	American Type Culture Collection (ATCC)	#CRL-3216
Human: HEK293 cells	American Type Culture Collection (ATCC)	#CRL-1573
Human: HIBCPP cells	Ishiwata et al.^50^	N/A
Human: HEK293 ADAM10/17 ^−/−^	Riethmueller et al.^62^	N/A
Human: HEK293_hGPR125	This paper	N/A

**Experimental models: Organisms/strains**		

*Xenopus laevis* oocytes	EcoCyte, Germany and this paper	N/A

**Recombinant DNA**		

hGPR125 FL_Flag (N-term.) _V5V5 (C-term.)_pcDNA3.1+ vector	Genscript	N/A
hGPR124 FL_Flag (N-term.) _V5V5 (C-term.) _pcDNA3.1+ vector	Genscript	N/A
hGPR125 CTF_artificial signal peptide (MKTIIALSYIFCLVFA)_ V5V5 (C-term.) _pcDNA3.1+ vector	Genscript	N/A
hGPR125 NTF_Flag (N-term.) pcDNA3.1+ vector	Genscript	N/A
hGPR125 FL_mCherry (N-term.) _eGFP (C-term.) _pcDNA3.1+ vector	Genscript	N/A
hGPR125 CTF_eGFP (C-term.) _pcDNA3.1+ vector	Genscript	N/A
hGPR125 FL_Flag (N-term.) α-bungarotoxin binding-site (ECL2) _pcDNA3.1+ vector	Genscript	N/A
hGPR125 FL GPS wt _Flag (N-term.) _V5V5 (C-term.)_pXOOM vector	Genscript	N/A
hGPR125 FL GPS mutation-> STKALS_Flag (N-term.) _Strep-tag (C-term.)_pXOOM vector	Genscript	N/A
hGPR125 FL GPS mutation-> ALS_Flag (N-term.) _Strep-tag (C-term.)_pXOOM vector	Genscript	N/A
hGPR125 FL GPS mutation-> HLA_Flag (N-term.) _Strep-tag (C-term.)_pXOOM vector	Genscript	N/A
hGPR125 FL GPS mutation-> NLS_Flag (N-term.) _Strep-tag (C-term.)_pXOOM vector	Genscript	N/A
hGPR125 FL GPS mutation-> QLS_Flag (N-term.) _Strep-tag (C-term.)_pXOOM vector	Genscript	N/A
hGPR125 FL GPS mutation-> ALA_Flag (N-term.) _Strep-tag (C-term.)_pXOOM vector	Genscript	N/A
hGPR125 FL GPS mutation-> RLS_Flag (N-term.) _Strep-tag (C-term.)_pXOOM vector	Genscript	N/A
hGPR125 FL GPS mutation-> SLD_Flag (N-term.) _Strep-tag (C-term.)_pXOOM vector	Genscript	N/A
hGPR125 FL GPS mutation-> SLG_Flag (N-term.) _Strep-tag (C-term.)_pXOOM vector	Genscript	N/A
hGPR125 FL GPS mutation-> SLH_Flag (N-term.) _Strep-tag (C-term.)_pXOOM vector	Genscript	N/A
hGPR125 FL GPS mutation-> SLN_Flag (N-term.) _Strep-tag (C-term.)_pXOOM vector	Genscript	N/A
hGPR125 FL GPS mutation-> SLP_Flag (N-term.) _Strep-tag (C-term.)_pXOOM vector	Genscript	N/A
hGPR125 FL GPS mutation-> SLQ_Flag (N-term.) _Strep-tag (C-term.)_pXOOM vector	Genscript	N/A
hGPR125 FL GPS mutation-> SLR_Flag (N-term.) _Strep-tag (C-term.)_pXOOM vector	Genscript	N/A
hGPR125 FL GPS mutation-> STKAVS_Flag (N-term.) _Strep-tag (C-term.)_pXOOM vector	Genscript	N/A
hGPR125 FL GPS mutation-> HLS_Flag (N-term.) _Strep-tag (C-term.)_pXOOM vector	Genscript	N/A
hGPR125 FL GPS mutation-> SAS_Flag (N-term.) _Strep-tag (C-term.)_pXOOM vector	Genscript	N/A
hGPR125 FL GPS mutation-> SLP_Flag (N-term.) _V5V5-tag (C-term.)_pXOOM vector	Genscript	N/A
hGPR125 FL GPS mutation-> SLR_Flag (N-term.) _V5V5-tag (C-term.)_pXOOM vector	Genscript	N/A
hGPR125 FL GPS mutation-> SLH_Flag (N-term.) _V5V5-tag (C-term.)_pXOOM vector	Genscript	N/A
hGPR125 FL GPS mutation-> HLT_Flag (N-term.) _V5V5-tag (C-term.)_pXOOM vector	Genscript	N/A
hGPR125 FL GPS mutation-> SLP_Flag (N-term.) _V5V5-tag (C-term.)_pcDNA3.1+ vector	Genscript	N/A
hGPR125 FL GPS mutation-> SLR_Flag (N-term.) _V5V5-tag (C-term.)_pcDNA3.1+ vector	Genscript	N/A
hGPR125 FL GPS mutation-> SLH_Flag (N-term.) _V5V5-tag (C-term.)_pcDNA3.1+ vector	Genscript	N/A
hGPR125 FL GPS mutation-> HLT_Flag (N-term.) _V5V5-tag (C-term.)_pcDNA3.1+ vector	Genscript	N/A
hGPR125 FL GPS mutation-> AAA_Flag (N-term.) _V5V5-tag (C-term.)_pcDNA3.1+ vector	Genscript	N/A
mGPR125 FL_FlagFlag (N-term.) _V5V5 (C-term.) pcDNA3.1+ vector	Genscript	N/A
rGPR125 FL_FlagFlag (N-term.) _V5V5 (C-term.) pcDNA3.1+ vector	Genscript	N/A
mGPR125 NTF_rGPR125CTF_FlagFlag (N-term.) _V5V5 (C-term.) pcDNA3.1+ vector	Genscript	N/A
zGPR125 FL_FlagFlag (N-term.) _V5V5 (C-term.)_pcDNA3.1+ vector	Genscript	N/A
hGPR125 aa 700 I_Flag (N-term.) _V5V5 (C-term.)_ pcDNA3.1+ vector	Genscript	N/A
hGPR125 aa 718 AAA_Flag (N-term.) _V5V5 (C-term.)_ pcDNA3.1+ vector	Genscript	N/A
rGPR125 aa 679 V, aa 684 I_FlagFlag (N-term.) _V5V5 (C-term.)_ pcDNA3.1+ vector	Genscript	N/A
rGPR125 aa700 V_FlagFlag (N-term.) _V5V5 (C-term.)_ pcDNA3.1+ vector	Genscript	N/A
rGPR125 aa 679 V, aa 684 I, aa 700 V_FlagFlag (N-term.) _V5V5 (C-term.)_ pcDNA3.1+ vector	Genscript	N/A
Klotho_ V5 (C-term.)_pcDNA3.1+ vector	Genscript	N/A

**Software and algorithms**		

Fiji (ImageJ)	Fiji developers	Version: 2.16.0/1.54p, https://fiji.sc
LICOR Image Studio 6.0	LI-COR Image Studio Software	RRID:SCR_015795
GraphPad Prism 10	GraphPad Software, LLC.	RRID:SCR_002798
Geneious Prime	Geneious Prime Software, Inc	RRID:SCR_010519
NetOGlyc-4.0	Center for Biological Sequence Analysis (CBS), Technical University of Denmark (DTU).	RRID:SCR_009026
SignalIP-4.1 and SignalIP-5.0	Center for Biological Sequence Analysis (CBS), Technical University of Denmark (DTU).	N/A
DataAnalysis 4.0	Bruker Daltonics	N/A
AxioVision Rel 4.8 acquisition software	Carl Zeiss Microscopy GmbH	RRID:SCR_002677
BioRender	BioRender Inc.	RRID:SCR_018361
ColabFold	ColabFold	RRID:SCR_025453
AlphaFold2	DeepMind	RRID:SCR_025454
UniProtKB	UniProt Consortium	RRID:SCR_004426
Maestro BioLuminate (Release 2020-3)	Schrödinger, Inc.	N/A
Prime	Schrödinger, Inc.	RRID:SCR_014887
Epik	Schrödinger, Inc.	RRID:SCR_016745
VSGB (Variable Surface Generalized Born) solvent model	Schrödinger, Inc.	N/A
OPLS3e force field	Schrödinger, Inc.	N/A
PeptideProphet	Institute for Systems Biology (ISB), Seattle	RRID:SCR_000274
IonQuant (v.1.10.27)	Nesvilab, University of Michigan	N/A
MSFragger (v.22)	Nesvilab, University of Michigan	N/A
Philosopher (v.5.1.1)	Nesvilab, University of Michigan	N/A

**Other**		

Lipofectamine 2000	Invitrogen	#11668–019
PET inserts, pore size: 5 μm	Sarsted	#83.3932.500

### Experimental model and study participant details

#### Cell lines and maintenance

Human embryonic kidney-293 (HEK 293T) cells derived from a female donor (American Type Culture Collection) were maintained in DMEM containing GlutaMAX (Gibco) supplemented with 10% fetal bovine serum (FBS), penicillin (180 U/ml), and streptomycin (45 μg/mL), and incubated at 37°C with 5% CO_2_ and 95% air humidity.

ADAM10/17-deficient HEK293 cells were kindly provided by Prof. Christoph Garbers, Medizinische Hochschule Hannover.^62^ Cells were maintained in DMEM containing GlutaMAX (Gibco) supplemented with 10% FBS, penicillin (180 U/ml), and streptomycin (45 μg/mL), and incubated at 37°C with 5% CO_2_ and 95% air humidity.

Pools of hGPR125 tetracycline-inducible HEK293T cell clones were maintained in DMEM containing GlutaMAX (Gibco) supplemented with 10% FBS (Sigma-Aldrich), 1% penicillin (180 U/ml), and streptomycin (45 μg/mL), 150 μg/mL hygromycin (ThermoFisher Scientific), 15 μg/mL blasticidine (ThermoFisher Scientific), and incubated at 37°C with 5% CO_2_ and 95% air humidity. To induce hGPR125 production, 50–100 ng/mL tetracycline was added to the cells.

Human choroid plexus papilloma (HIBCPP) cells, derived from a female donor, were maintained in DMEM/F12, 10% v/v heat-inactivated FBS (Gibco), 5 μg/mL human insulin (Sigma Aldrich), 4 mM L-Glutamine (Gibco), 100 μg/mL streptomycin, and 100 U/ml penicillin (Gibco) (HIBCPP-10 medium).

#### Mycoplasma test

All cell lines were tested for mycoplasma using the MycoAlert PLUS Mycoplasma Detection Kit (Lonza) or the Mycoplasma PCR Detection Kit (ScienCell).

#### Cerebrospinal fluid sample collection and storage

The CSF data used in this work was generously provided by participants under the HD-Clarity and HD-CSF cerebrospinal fluid collection initiatives (Lead PI Ed Wild, sponsored by University College London) and were made available by CHDI Foundation, Inc. The Medical Research Council UK (MR/M008592/1) funded HD-CSF. HD-Clarity is a study within Enroll-HD, a global clinical research platform for Huntington’s disease, funded by CHDI Foundation, Inc. HDClarity (ClinicalTrials.gov ID NCT02855476) is performed in accordance with the principles of the Declaration of Helsinki, and the International Conference on Harmonization and the World Health Organization Good Clinical Practice standards. All participating sites sought appropriate ethical approval in accordance with each specific country legislation. All reported participants gave informed consent prior to undertaking study procedures.

CSF were collected from 131 mixed sex healthy participants (age ranging from 23 to 75 with an average of 49 years old) as part of the CHDI HD-Clarity study. Samples were collected and processed according to a standard protocol.^75^ After processing, samples were immediately stored at −80°C until distribution to investigators.^76^ Samples from both sexes were included in the CSF cohort; however, the study was not powered or designed to assess sex-dependent differences, and no stratified sex-based analyses were performed. No experimental group allocation was performed because all CSF samples were analyzed as an observational cohort for detection of GPR125-derived peptides.

### Method details

#### Recombinant DNA

The following recombinant DNA constructs were custom made by Genscript

• hGPR125 FL_Flag (N-term.) _V5V5 (C-term.) _pcDNA3.1+ vector
• hGPR124 FL_Flag (N-term.) _V5V5 (C-term.) _pcDNA3.1+ vector
• hGPR125 CTF_artificial signal peptide (MKTIIALSYIFCLVFA)_ V5V5 (C-term.) _pcDNA3.1+ vector
• hGPR125 NTF_Flag (N-term.) pcDNA3.1+ vector
• hGPR125 FL_mCherry (N-term.) _eGFP (C-term.) _pcDNA3.1+ vector
• hGPR125 FL_Flag (N-term.) α-bungarotoxin binding-site (ECL2) _pcDNA3.1+ vector
• hGPR125 FL GPS mutation STKALS_Flag (N-term.) _Strep-tag (C-term.)_pXOOM vector
• hGPR125 FL GPS mutation ALS_Flag (N-term.) _Strep-tag (C-term.)_pXOOM vector
• hGPR125 FL GPS mutation HLA_Flag (N-term.) _Strep-tag (C-term.)_pXOOM vector
• hGPR125 FL GPS mutation NLS_Flag (N-term.) _Strep-tag (C-term.)_pXOOM vector
• hGPR125 FL GPS mutation QLS_Flag (N-term.) _Strep-tag (C-term.)_pXOOM vector
• hGPR125 FL GPS mutation ALA_Flag (N-term.) _Strep-tag (C-term.)_pXOOM vector
• hGPR125 FL GPS mutation RLS_Flag (N-term.) _Strep-tag (C-term.)_pXOOM vector
• hGPR125 FL GPS mutation SLD_Flag (N-term.) _Strep-tag (C-term.)_pXOOM vector
• hGPR125 FL GPS mutation SLG_Flag (N-term.) _Strep-tag (C-term.)_pXOOM vector
• hGPR125 FL GPS mutation SLH_Flag (N-term.) _Strep-tag (C-term.)_pXOOM vector
• hGPR125 FL GPS mutation SLN_Flag (N-term.) _Strep-tag (C-term.)_pXOOM vector
• hGPR125 FL GPS mutation SLP_Flag (N-term.) _Strep-tag (C-term.)_pXOOM vector
• hGPR125 FL GPS mutation SLQ_Flag (N-term.) _Strep-tag (C-term.)_pXOOM vector
• hGPR125 FL GPS mutation SLR_Flag (N-term.) _Strep-tag (C-term.)_pXOOM vector
• hGPR125 FL GPS mutation STKAVS_Flag (N-term.) _Strep-tag (C-term.)_pXOOM vector
• hGPR125 FL GPS mutation HLS_Flag (N-term.) _Strep-tag (C-term.)_pXOOM vector
• hGPR125 FL GPS mutation SAS_Flag (N-term.) _Strep-tag (C-term.)_pXOOM vector
• hGPR125 FL GPS mutation SLP_Flag (N-term.) _V5V5-tag (C-term.)_pXOOM vector
• hGPR125 FL GPS mutation SLR_Flag (N-term.) _V5V5-tag (C-term.)_pXOOM vector
• hGPR125 FL GPS mutation SLH_Flag (N-term.) _V5V5-tag (C-term.)_pXOOM vector
• hGPR125 FL GPS mutation HLT_Flag (N-term.) _V5V5-tag (C-term.)_pXOOM vector
• hGPR125 FL GPS mutation SLP_Flag (N-term.) _V5V5-tag (C-term.)_pcDNA3.1+ vector
• hGPR125 FL GPS mutation SLR_Flag (N-term.) _V5V5-tag (C-term.)_pcDNA3.1+ vector
• hGPR125 FL GPS mutation SLH_Flag (N-term.) _V5V5-tag (C-term.)_pcDNA3.1+ vector
• hGPR125 FL GPS mutation HLT_Flag (N-term.) _V5V5-tag (C-term.)_pcDNA3.1+ vector
• hGPR125 FL GPS mutation AAA_Flag (N-term.) _V5V5-tag (C-term.)_pcDNA3.1+ vector
• rat rGPR125 FL_FlagFlag (N-term.) _V5V5 (C-term.) pcDNA3.1+ vector
• mGPR125 NTF_rGPR125CTF_FlagFlag (N-term.) _V5V5 (C-term.) pcDNA3.1+ vector
• zGPR125 FL_FlagFlag (N-term.) _V5V5 (C-term.)_pcDNA3.1+ vector
• hGPR125 aa 700 I_Flag (N-term.) _V5V5 (C-term.)_ pcDNA3.1+ vector
• hGPR125 aa 718 AAA_Flag (N-term.) _V5V5 (C-term.)_ pcDNA3.1+ vector
• rGPR125 aa 679 V, aa 684 I_FlagFlag (N-term.) _V5V5 (C-term.)_ pcDNA3.1+ vector
• rGPR125 aa700 V_FlagFlag (N-term.) _V5V5 (C-term.)_ pcDNA3.1+ vector
• rGPR125 aa 679 V, aa 684 I, aa 700 V_FlagFlag (N-term.) _V5V5 (C-term.)_ pcDNA3.1+ vector
• Klotho_ V5 (C-term.)_pcDNA3.1+ vector

#### Transient transfection of cells

HEK293T cells were transfected using Lipofectamine 2000 (Invitrogen) according to the manufacturer’s protocol. 24h before transfection, 4 × 10^5^ cells were seeded into tissue culture-treated six-well plates, and on the following day transfected with 1 μg of DNA. The empty pcDNA3.1+ vector was used as a vector control.

#### Generation of the hGPR125 expressing HEK293 cell line

HEK293T cells were transfected as described above. The transfected cells were selected with G418 (400 μg/mL) 24h after initial transfection. Cells were detached with 0.5 mL trypsin-EDTA resuspended in 2 mL selection medium and plated onto 6-well plates at serial dilutions (1/25, 1/50, 1/100, 1/250, 1/500). A “dead-control” flask (non-transfected cells) was maintained in parallel under identical selection pressure. After approximately 4–5 days of exposure to G418, non-resistant cells in the dead-control flask displayed progressive cell death, confirming selection efficacy. Resistant colonies were identified by light microscopy. To isolate individual colonies, wells were washed with PBS, and a sterile cloning cylinder was positioned over the selected colony. 50 μL trypsin-EDTA was added inside the cylinder for ∼60 s. Cells were gently resuspended and transferred to a fresh T25 flask containing selection medium. Successful transfer was confirmed microscopically. Isolated clones were expanded in T25 flasks with medium changes every 2–3 days. Once confluent, clones were further passaged into T75 or T175 flasks as required. GPR125 protein expression was confirmed by Western Blotting.

#### Mass spectrometry and proteomic data analysis of human cerebrospinal fluid samples

Human cerebrospinal fluid samples were processed in a sodium deoxycholate (SDC) buffer (1% w/v SDC; 40 mM 2-Chloroacetamide, “CAA; 10 mM Tris(2-carboxyethyl) phosphine hydrochloride, “TCEP; 100 mM Tris, all Sigma-Aldrich in MS grade water, VWR) and boiled at 95°C for 10 min at 1200rpm to denature proteins. Trypsin (T6567; Sigma-Aldrich) and LysC (125–02543; Fujifilm, WAKO) enzymes were added at 1:100 (w/w) for protein digestion for 2 h at 37°C and 800rpm. The reaction was quenched by acidification using quenching buffer (MS grade isopropanol, Fisher Scientific; 1% w/w trifluoroacetic acid, “TFA”, Sigma-Aldrich). The peptides were then cleaned on Stagetips containing two layers of SDB-RPS material (Empore) through two organic buffer wash steps (quenching buffer, described above) and two inorganic buffer wash steps (0.2% TFA, Sigma-Aldrich in MS grade water, VWR), and peptides were then eluted using a basic elution buffer (1% ammonia and 80% MS grade acetonitrile, both Merck in MS grade water, VWR) and vacuum-dried to complete dryness at 45°C. Dry peptides were then stored at −80°C until shortly before the samples were scheduled for mass spectrometry analysis, at which time they were resuspended in a mass spectrometry compatible buffer (0.1% TFA, Sigma-Aldrich; 5% MS grade acetonitrile, Merck in MS grade water, VWR). 400 ng peptides per sample were loaded onto Evotips according to the manufacturer’s instructions (Evotip Quick Guide, 2022 version) using isopropanol (MS grade isopropanol, Fisher Scientific), Evosep solvent A (0.1% formic acid, Merck in milliQ water) and Evosep solvent B (0.1% formic acid in MS grade acetonitrile, Merck). Samples were injected on an Astral Orbitrap mass spectrometer (Thermo Fisher Scientific) connected to an Evosep One HPLC system (Evosep Biosystems) by an 80 mm analytical column (EV1115 Performance column, Evosep Biosystems) kept at 55°C and attached to an EASY-Spray ion source. Peptides were eluted using a standardized 21-min gradient, and all samples were run in data independent acquisition (DIA) mode with the instrument operated at a full-MS resolution of 240,000 with a full scan range of 380–980 m/z with automatic gain control (AGC) target set to 500%, and a maximum injection time (IT) of 3 ms. Fragment ion (MS/MS) scans ran with 200 3-Th windows with a scan range of 150–2000 m/z and a maximum IT of 5 ms. Isolated ion fragmentation was conducted using high-energy collision-induced dissociation (HCD) at 25% normalized collision energy (NCE). A standardized HeLa digest and a pooled cerebrospinal fluid digest were used as technical and internal quality controls, respectively, and were acquired at regular intervals to ensure consistent instrument performance and sample reproducibility throughout. Raw files were processed in Spectronaut version 20.1 (Biognosys) using directDIA in a two-step process wherein a spectral library was created from multiple search archives and used to analyze the full dataset to ease computational load. Label-free quantification (LFQ) was used to estimate relative protein abundances across samples. For analysis, enzyme specificity was set to Trypsin/P with peptides from 7 to 52 amino acid lengths and maximum 2 missed cleavages allowed, and carbamidomethyl (C) was permitted as a fixed modification, and oxidation (M) and acetylation (N-term) were permitted as variable modifications. The false discovery rate (FDR) was set to 0.01 on all levels, and cross-run normalization was run in global mode. Run-level protein scoring was performed by highest scoring observation. All bioinformatic analyses were conducted in Python using the Clinical Knowledge Graph (CKG).^77^ As part of quality control, hemoglobin related markers (HBA2, HBB, HBD) were evaluated across all samples using standardized z-scores to identify potential outliers and to assess whether extreme values (|*Z* score >3|) influenced the total number of detected proteins, as previously described. GPR125 was identified by 4 tryptic peptides. These peptides were not detected across all samples. RAYLAYR, LAGGAHVYSTYSPNIALEAYVIK, IGCLNADIFR, and NTHGSGIYPGNPQDER were identified across the samples with a data completeness of 24%, 18%, 13% and 5%, respectively.

#### Cell viability assay

HEK293T cells were transiently transfected either FL FLAG-GPR125-V5V5 or CTF GPR125 coding plasmids, in doses of 50, 25, 5 or 0 (mock) ng DNA per 30 000 cells. Differences in receptor DNA used for transfection were filled with vector DNA (pcDNA3.1+, Genscript) to the total amount of 50 ng per 30 000 cells. Transfection was performed using Lipofectamine 2000 in Opti-MEM I Reduced-Serum Medium, according to the manufacturer’s protocol (ThermoFisher Scientific). In parallel, to obtain ncNTF-conditioned medium and its background control, confluent flasks of HEK293 cells stably expressing FL FLAG-GPR125-V5V5 and parental HEK293 cells were incubated with serum-free medium (15 mL/T175) for 24 h. On the next day, the medium from the overnight culture was harvested, centrifuged, and applied on the transfected HEK293T cells for another 24 h. Additionally, cells incubated with 100% lysis buffer (M-PER Mammalian Protein Extraction Reagent, ThermoFisher Scientific) were used as a negative control. On the following day, the cell viability assay was performed with the AlamarBlue Cell Viability Reagent (detecting living cells based on the activity of mitochondrial reductases) according to the manufacturer’s protocol (ThermoFisher Scienific). Mock-transfected cells incubated in serum-free medium harvested from parental HEK293 cells (without ncNTF) were referred as 100% viability sample, while the cells treated overnight with the lysis buffer were referred as 0% viability sample. The data were collected from five independent experiments, performed in technical duplicates, and presented as the means of technical replicates for each experiment. Statistically significant differences in viability were verified with two-way ANOVA and Fisher’s Least Significant Difference (LSD) post-hoc test and depicted between ncNTF-stimulated and non-stimulated cells transfected with the same amount and type of receptor DNA for ∗∗∗∗*p* < 0.0001.

#### Proteomics identifying cleavage site of GPR125

Concentrated media samples from transfected and non-transfected HEK cells and cell lysate from transfected HEK cells were treated with PNGase F to remove attached sugars. Samples were reduced with tris(2-carboxyethyl) phosphine (TCEP, 10 mM) and alkylated using 2-chloroacetamide (CAA, 40 mM) before undergoing SP3 clean-up and on bead digestion as previously described.^78^ Proteins were resuspended in either 50 mM TEAB (Trypsin and Glu-C), 50 mM TEAB, 2 mM CaCl_2_, 0.1 mM MgCl_2_ (TrypN) and digested with Trypsin (Promega, V5111), TrypN (Protifi, K001) or GluC (Promega, V1651) at a 1:50 enzyme to protein ratio for 16h at 37°C. Peptides were desalted with P200 tips packed with two C18 disks (Affinisep) and 1/10^th^ of the sample was removed to a new tube and kept for full proteome analysis, with the remainder used for N- and C-terminal enrichment. For the latter, P200 tips were loaded with two strong cation exchange disks (Empore) and conditioned for N-/C-terminal enrichment as previously described.^79^ The flow-through from sample loading and elution was combined into one fraction which was dried down and termed the ‘enriched fraction’ with the trypsin digested samples resulting in a C-terminal enrichment and the TrypN digested samples resulting in an N-terminal enrichment. All samples were reconstituted in 0.1% TFA and analyzed using a Dionex Ultimate 3000 RSLCnano system (Thermo Scientific) with an Aurora reversed phase C18 column (15 cm × 75 μm i.d., 1.6 μm C18, IonOpticks), maintained at 35°C, coupled to a timsTOF Pro (Bruker). Fragpipe was used to facilitate database searching with MSFragger (v.22) against the human proteome (UP000005640, downloaded September 2024) with cysteine alkylation as a fixed modification and a peptide length between 7–50 residues, a maximum of two missed cleavages, and N-terminal acetylation and methionine oxidation as variable modifications allowed (standard settings). Validation was conducted using MSbooster and Percolator with quantification conducted through IonQuant (v. 1.10.27) with match between runs enabled. MS/MS spectra were visualized using FragPipe-PDV viewer. The schematic overview of the GPR125 ncNTF indicating the identified peptides was generated using the GPCR database.^80^

#### Using whole cell lysates

Cells were transfected in 6-well plates with the indicated constructs or empty vector. 48h after transfection, cells were put on ice, washed with Phosphate Buffered saline with Ca and Mg (PBS^++^) and scraped off with 100 μL RIPA buffer containing protease (Roche) and phosphatase (Sigma-Aldrich) inhibitors, transferred to 1.5 mL tubes, and incubated for 30 min at 4°C on a spinning disc with gentle rotation. Lysates were cleared by centrifugation, and protein concentration was measured using a bicinchoninic acid (BCA) assay kit (Pierce).

#### Using cell medium of transfected cells

Cells were transfected in 6-well plates with the indicated constructs or empty vector. 48h before harvest, cell medium was changed to Opti-MEM to reduce the amount of protein in the cell medium. Medium was collected and cleared by centrifugation at 10,000 g for 10 min at 4°C, then ∼10x concentrated using 30 K concentrators (Amicon/Vivaspin). The protein content of the concentrated cell medium was measured using a BCA assay kit (Pierce).

#### Testing protease inhibitors on GPR125 ncNTF

GPR125-overexpressing HEK293 cells were seeded on 6-well plates (3 × 10^5^ cells/well) and cultured overnight. Next, the growth medium was replaced with Opti-Mem supplemented with tetracycline (1 μg/ml, Sigma-Aldrich), and the following protease inhibitors were added to some wells: (i) GW280264X (10 μM, Tocris), an inhibitor of ADAM17 and ADAM10; (ii) GI 254023X (10 μM, Sigma-Aldrich), an inhibitor of ADAM10; (iii) batimastat (10 μM, Tocris), a broad spectrum metalloprotease inhibitor; (iv) 0664283 (10 μM, Sigma-Aldrich), a general inhibitor of BACE. DMSO, as vehicle used to dissolve inhibitors was added to control wells. Cells were cultured in the presence of inhibitors for 48 h. Next, culture medium was collected and cleared by centrifugation at 10,000 g for 10 min at 4°C, then concentrated down to ∼20 μL using 30 K concentrators (Amicon/Vivaspin) and analyzed by western blotting. Cells were lysed as described in **Using whole cell lysates** and the cell lysates were subjected to western blot analysis.

#### Insulin effects on Klotho and ncNTF shedding

After transient transfection, cells were kept in DMEM containing GlutaMAX (Gibco) supplemented with 10% FBS, 1% penicillin (180 U/ml), and streptomycin (45 μg/mL), and incubated at 37°C with 10% CO_2_. 48 h post-transfection, medium was removed from the cells and cells were carefully washed with PBS. Fresh serum-free DMEM was added to the cells (1 mL per well, 6-well plate) containing no insulin for the control groups or containing 1 μM insulin (Lonza). Cells were incubated for ∼6 h at 37°C with 5% CO_2_. Medium was collected and cleared by centrifugation at 10,000 g for 10 min at 4°C. Medium corresponding to three wells (3 mL) was concentrated down to ∼20 μL using 30 K concentrators (Amicon/Vivaspin) and analyzed by western blotting.

#### Western blot analysis

For each sample, 20–100 μg of cell lysate or concentrated cell medium were aliquoted and supplemented with 4x Protein Sample Loading Buffer (30,3 g/L tris, 400 mL/L glycerol 87%, 120 g/L SDS, 240 mg/L bromophenol blue, pH 6,8) containing the final concentration of 50 mM DTT. Samples were incubated for 30 min at 37°C prior to loading. Criterion™ TGX™ precast gels, 4–15% (BIO-RAD) were used for electrophoresis. A pre-stained protein ladder (BIO-RAD) was used as a molecular mass marker. The separated proteins were transferred to PVDF membranes (BIO-RAD) and blocked with Intercept TBS blocking buffer (LI-COR).

Antibodies were diluted in TBS with 0.1% Tween 20 (TBST) at the indicated concentrations: mouse anti-V5 (1:5000; Invitrogen), mouse anti-FlagM1 (1:3333; Sigma-Aldrich), mouse anti-FlagM2 (1:6666; Sigma-Aldrich), rabbit anti-GPR125 NTF (1:1000; Atlas antibodies), mouse anti-mCherry (1:2000; Sigma-Aldrich); mouse anti-eGFP (1:1000; Invitrogen) chicken anti-GAPDH (1:10000; Sigma-Aldrich), goat anti-mouse (1:10000, LI-COR), goat anti-rabbit (1:10000, LI-COR), donkey anti-chicken (1:10000, LI-COR).

Blots were incubated with the primary antibody overnight at 4°C and with the secondary antibody for 1h at room temperature. After antibody incubations, blots were washed 3x with TBST for 10 min each and thereafter briefly washed in TBS. For the anti-FlagM1 antibody, TBST/TBS supplemented with 1 mM CaCl_2_ was used. Blots were visualized using an LI-COR Odyssey Fc scanner.

For the western blots of the cell medium samples, protein-free blocking buffer (LICOR) was used to allow whole protein staining using the fluorescent Revert700 (LICOR) stain after immunoblotting. The staining was conducted according to the manufacturer’s instructions.

#### Immunofluorescent staining of fixed cells

HEK293T cells were seeded on glass inserts (Marienfeld) in 12-well plates at 100,000 cells/well in HEK293T cell medium. Cells were transfected with 0.5 μg DNA 24 h after cell seeding. 48 h after transfection, cells were fixed for 15 min in 4% paraformaldehyde, blocked with HBSS+ buffer containing 2% BSA, and permeabilized in HBSS+ buffer containing 0.1% Triton X-100 where indicated. Antibodies were diluted in HBSS+ at the indicated concentrations: mouse anti-FlagM1 (1:800; Sigma-Aldrich), rabbit anti-GPR125 NTF (1:50; Atlas antibodies), goat anti-mouse (1:1000; Jackson ImmunoResearch), goat anti-rabbit (1:1000; Jackson ImmunoResearch). Hoechst (10 μg/mL; Thermofisher Scientific) was used for nuclear counterstaining, and sections were mounted with Pro-Long Diamond antifade reagent (invitrogen). Images were acquired with a Zeiss Axiovert 200M (Carl Zeiss AG, Oberkochen, Germany) and AxioVision Rel 4.8 acquisition software (Carl Zeiss AG) using a 63x objective and processed in Fiji ImageJ.

HIBCPP cells at passages 22–26 were seeded on inverted PET filter inserts of 0.33 cm^2^ size with 5 μm pores size (Sarstedt) at 100,000 cells/filter in HIBCPP-10 medium as described previously.^81^ Filter inserts were brought back to a hanging position 24 h later (day 1). On day 3 medium was changed to a medium containing only 1% FBS. On day 5 filter inserts were fixed in MeOH at −20°C for 20 s or in 4% paraformaldehyde for 15 min at room temperature, respectively, followed by blocking and staining in blocking buffer (5% skimmed milk, 0.3% Triton X-100 in Tris-buffered saline) with goat anti-GPR125 antibody targeting the CTF (25 μg/mL, Novus Biologicals, NBP1-03561) or rabbit anti-GPR125 antibody targeting the (nc)NTF (2 μg/mL, Atlas antibodies, HPA008904), respectively, and mouse anti-E-Cadherin antibody (0.8 μg/mL, BD biosciences) over night at 4°C. Normal goat or normal rabbit IgG control (both from R&D Systems) were used as isotype controls and Alexa fluor 488-conjugated anti-goat (Invitrogen) or Alexa fluor A647-conjugated anti-rabbit and Cy3-conjugated donkey anti-mouse (Jackson Immunology Research) as secondary antibodies. Filter inserts were mounted with mowiol (Sigma-Aldrich). Images of HIBCPP cells for z-stacks were acquired with an LSM 800 confocal microscope (Zeiss) with a 63x objective and processed in Fiji ImageJ.

#### Live-cell microscopy

HEK293T cells were seeded in Nunc 8-well chamber slides (ThermoFisher Scientific Cat#154534 PK) at 20,000 cells/well in HEK293T cell medium. Cells were transfected with 0.21 μg DNA 24 h after cell seeding. 48 h after transfection, cells were placed on ice and stained with α-bungarotoxin, Alexa Fluor™ 488 conjugate (1:125), and Hoechst (10 μg/mL) for nuclear counterstaining. After 10 min, anti-FlagM1 (1:100) and Hoechst (10 μg/mL) was added. After 10 min, the cells were washed 2 times with 100 mL HBSS+CaCl_2_+MgCl_2_ and incubated with Alexa fluor 568-conjugated goat anti-mouse (1:100) and Hoechst (10 μg/mL). After 10 min, the cells were washed 2 times with 100 μL HBSS+CaCl_2_+MgCl_2_ and kept on ice until immediately before image acquisition. Images at timepoint 0 min were acquired at room temperature, after which the temperature in the microscopy heating chamber was turned up to 37°C to allow internalization. Images were acquired with Andor Dragonfly Spinning Disk confocal microscope using Fusion Software (Oxford Instruments, Abingdon, United Kingdom). Images were collected using a 63x objective and processed in Imaris and Fiji ImageJ.

#### Oocyte preparation and protein expression

The *Xenopus laevis* oocytes were surgically removed from *Xenopus laevis* frogs (Nasco, US) according to European Community guidelines for the use of experimental animals and under a license issued for the use of experimental animals by the Danish Ministry of Justice (Dyreforsøgstilsynet) or obtained from EcoCyte Bioscience, Dortmund, Germany. After surgical removal of the oocytes from anesthetized frogs, the follicular membrane was removed by incubation in Kulori medium (90 mM NaCl, 1 mM KCl, 1 mM MgCl_2_, 1 mM CaCl_2_, 5 mM HEPES, and ∼4 mM Tris (HOCH_2_)_3_CNH_2_ (pH 7.4)) containing 10 mg/mL collagenase (type 1; Worthington) and trypsin inhibitor (1 mg/mL; Sigma-Aldrich) for 1 h. Subsequently, the oocytes were washed five times in Kulori medium containing 0.1% bovine serum albumin (Sigma-Aldrich) and incubated in 100 mM K2HPO4 with 0.1% BSA for 1 h cDNA constructs of hGPR125 wt and GPS motif mutants in the expression vector pXOOM were obtained from Genscript, and the sequences were verified. The cDNA constructs were linearized downstream of the poly(A) segment and subsequently transcribed with the T7 mMessage Machine kit according to the instructions of the manufacturer (Ambion, Austin, TX). After transcription, the acquired cRNA was extracted using the MEGAclear (Ambion) kit prior to microinjection into defolliculated *X. laevis* oocytes (8–16 ng of cRNA/oocyte). The microinjected oocytes were kept in Kulori medium at 18°C for 72h before the experiments were performed.

#### Oocyte preparation for western blotting

*Xenopus laevis* oocytes expressing the hGPR125 wt and GPS motif mutants were homogenized by pipetting 72 h after RNA injection in 1 mL HbA buffer (5 mM MgCl_2_, 5 mM NaH_2_PO_4_, 1 mM EDTA, 80 mM sucrose, 20 mM Tris, pH 7.48) containing 8 μM leupeptin (Sigma) and 0.4 mM Pefabloc (Sigma) (HbA+). The homogenized oocytes were subsequently spun down for 10 min at 250 g, the supernatant was transferred to a new tube, spun down for 20 min at 16000 g and the cell pellet was resuspended in 5 μL/1.5 oocytes HbA+ buffer. All steps were carried out on ice or in a centrifuge cooled down to 4°C. For Western Blotting, 5–10 μL corresponding to 1.5–3 oocytes were supplemented with 4x Protein Sample Loading Buffer (30,3 g/L tris, 400 mL/L glycerol 87%, 120 g/L SDS, 240 mg/L bromophenol blue, pH 6,8) containing the final concentration of 50 mM DTT and incubated for 30 min at 37°C. Western Blot was carried out as described.

#### GAIN domain stability model

Human, rat and mouse ADGRA3 GAIN domain models were created using ColabFold^82^ based on AlphaFold2^83^ with sequences retrieved from UniProtKB truncated at the C-terminus of the annotated GPS motif, including the tethered agonist. For analysis of mutational impact, structures were imported into Maestro BioLuminate^84^ and prepared by adding hydrogen atoms, estimating residue protonation at pH 7.0 with Epik.^85^ The prepared structures were minimized using Prime^55^ with the implicit VSGB solvent model^56^ and the OPLS3e Force field.^57^ Via MAESTRO built-in residue scanning function, the impact of point mutations was predicted for the used GPS triad mutations of human GPR125 and the amino acids differing between rat and mouse GPR125 contained within the GAIN subdomain B within 100 residues upstream of the GPS triad. Mutational impact was estimated by using PRIME and MAESTRO, comprised differences in the PRIME energy function^56^ and a correction for solvent accessible surface area between unmutated and mutated structural model. These metrics were generated via refinement of side chain prediction with backbone sampling of residues with a 7.00 Å cutoff around mutated residues.

### Quantification and statistical analysis

#### Measuring mean fluorescent intensity (MFI)

Mean fluorescent intensity in the live-cell microscopy images was measured using the software Fiji/ImageJ. To calculate the ratio between the MFI in the cytoplasm and in the plasma membrane, multiple regions of interest (ROIs) were manually selected and measured at the plasma membrane or cytoplasm of ten cells per time point. The data were corrected by subtraction of the background signal in the images and plotted as mean ± SEM.

#### Western blots

Fluorescent western blots were analyzed using the Image Studio™ software. Protein bands were manually selected and measured by the program. For the cell medium samples, protein band signals were normalized to the whole protein stain (Revert 700) and where protein band intensities were compared across multiple lanes, intensities were normalized to GAPDH. For the cell medium and cell lysate samples used for the testing of the different protease inhibitors, a Coomassie whole protein stain was used for normalization. The Specified band intensity (%) over total band intensity per lane in the cell lysate blots was calculated as following:

$$FL\;\%:\frac{FL}{FL+ncCTF+{ncCTF}^{*}}*100$$

$$ncCTF\;\%:\frac{ncCTF}{FL+ncCTF+{ncCTF}^{*}}*100$$

$${ncCTF}^{*}\%:\frac{{ncCTF}^{*}}{FL+ncCTF+{ncCTF}^{*}}*100$$

$$ncNTF\%:\frac{ncNTF}{FL+ncNTF}*100$$

#### Statistical analysis

Statistical analyses were performed using GraphPad Prism (version 10.6.1). Depending on the experimental design, data were analyzed using unpaired two-tailed Student’s t-tests, one-way ANOVA, or two-way ANOVA. Homogeneity of variances was assessed using Brown-Forsythe or Bartlett’s tests for ANOVAs and an F-test for Student’s t-tests. ANOVAs were followed by Dunnett’s, Šídák’s, Tukey’s, or Fisher’s LSD post hoc multiple-comparisons tests, as indicated in the corresponding figure legends and Table S4. For the two-way ANOVA in Figure 1H, the interaction term was not significant (F(12,84) = 0.15, *p* = 0.9996); therefore, the final model was fitted without the interaction term, and the resulting main effects and Fisher’s LSD post hoc comparisons are reported. Effect sizes are reported as eta-squared (η^2^) for ANOVA models and as mean differences for pairwise comparisons. Differences were considered statistically significant at *p* ≤ 0.05. Unless otherwise indicated in the figure legends, data are presented as mean ± SEM; for Figure 1H, values from five independent experiments are shown individually. Information on biological and technical replicates and the statistical test used for each experiment is provided in the figure legends and Table S4.

## Supplemental information

Document S1. Figures S1–S8 and Tables S1–S3

Table S4. Statistical parameters for all quantitative analyses related to Figures 1–4 and Figures S5-S7
