# Supplementary material for: Non-canonical cleavage of GPR125/ADGRA3 releases extracellular receptor fragments detectable in cerebrospinal fluid
Source: iScience. 2026 Sep 21;29(10):117551. doi: 10.1016/j.isci.2026.117551 (PMC13631675; doi:10.1016/j.isci.2026.117551)
Supplement: Supplementary file 1 [file mmc1.pdf]

iScience, Volume *29*

Supplemental information

Non-canonical cleavage of GPR125/ADGRA3 releases extracellular receptor fragments detectable in cerebrospinal fluid

Anna L.S. Walser, Olav Larsen, Chiara Stüdle, Nicoline Wichmand Thorsen, Florian Seufert, Anna Katarzyna Drzazga, Renata Mężyk-Kopeć, Isha Raj, Filippa Liliendahl Qvist, Sofie Morsing Bagger, Gertrud Malene Hjortø, Trine Toft-Bertelsen, Emily Slowikowski, Silvia Bergt, Eva Ganseman, Christoffer Knak Goth, Juliane Lokau, Christoph Garbers, Hiroshi Ishikawa, Horst Schroten, Paul Proost, Anja Hviid Simonsen, Nicholas M.I. Taylor, Joanna Bereta, Nanna MacAulay, Peter W. Hildebrand, Niels Henning Skotte, Katja Spiess, Tobias Langenhan, Michael J. Davies, Britta Engelhardt, Pedro E. Marques, and Mette M. Rosenkilde

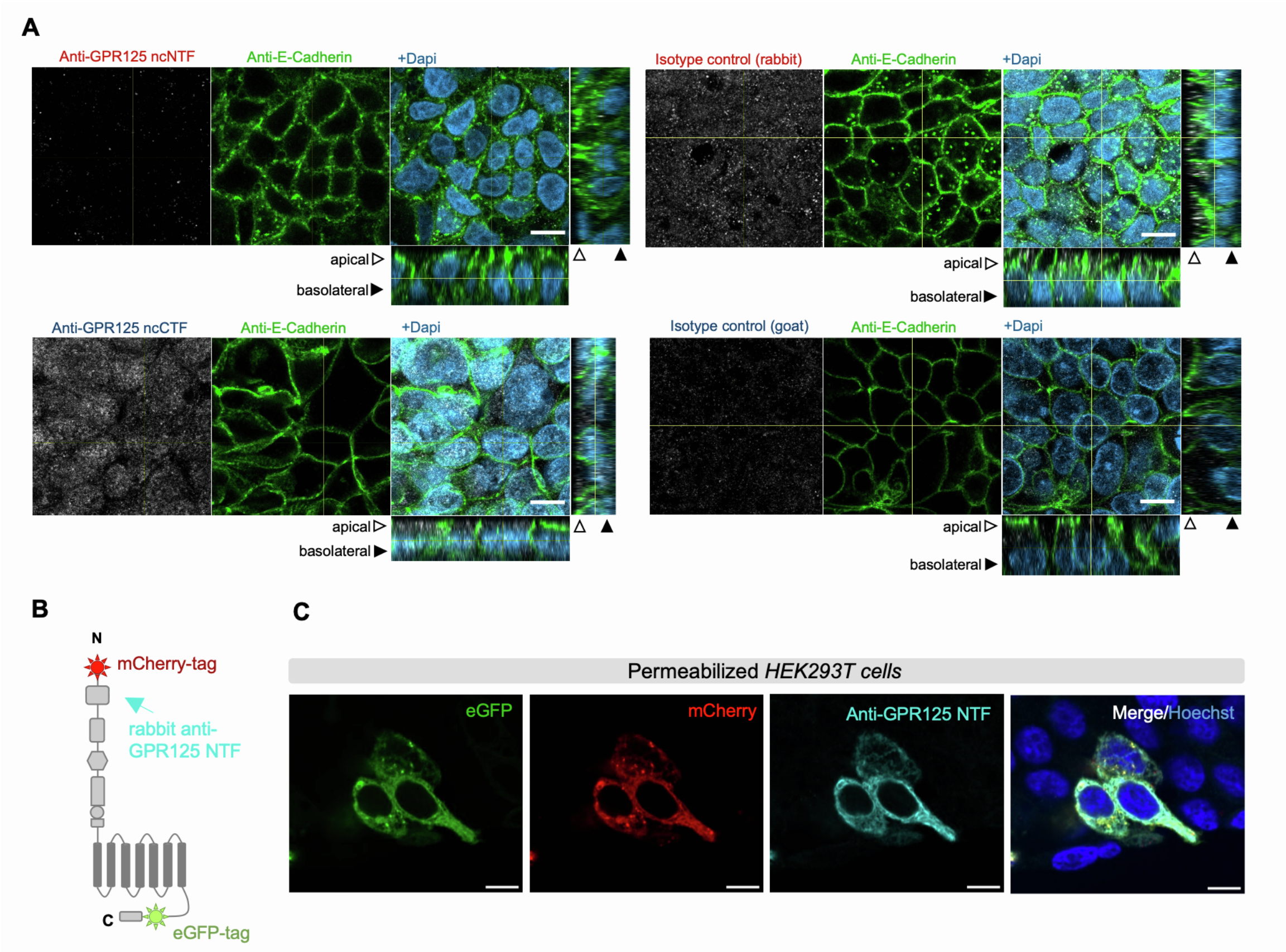

Figure S1. The anti-GPR125 ncCTF but not the anti-GPR125 ncNTF detects endogenous GPR125 in HIBCPP cells. ***Related to Figure 1.***

(A) Immunofluorescent staining of HIBCPP cells showing the anti-GPR125 ncNTF and ncCTF staining compared to the negative controls rabbit IgG (rbIgG) and goat IgG (goIgG).
The anti-E-cadherin, targeting an epithelial cell-cell adhesion molecule, has been included as a plasma membrane marker. Width of scale bar is 10 µm.
(B) Schematic overview of hGPR125 with an N-terminal mCherry-tag and a C-terminal eGFP-tag.
(C) Immunofluorescent staining of transfected HEK293T cells expressing hGPR125 shown in (B). The anti-GPR125 ncNTF detects hGPR125. Width of scale bar is 10 µm.

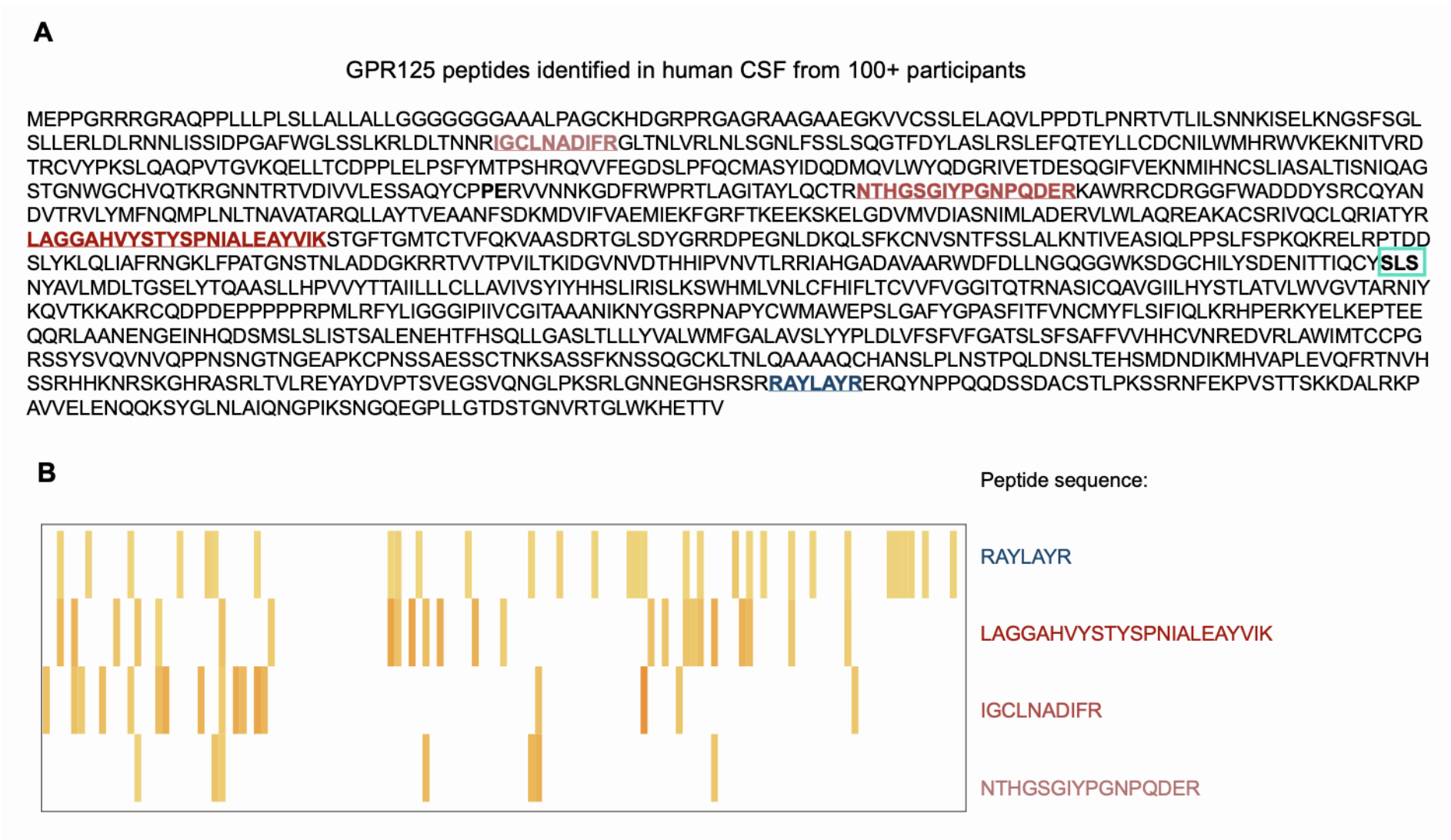

Figure S2. Data from MS analysis of human cerebrospinal fluid samples identifying GPR125 peptides. ***Related to Figure 1.***

MS analysis of human cerebrospinal fluid samples of 131 healthy participants. **(A)** GPR125 amino acid sequence highlighting the position of the identified tryptic GPR125 peptides. The GPS of GPR125 is highlighted with a green box.

(B) Overview of tryptic GPR125 peptides identified in the individual participants (yellow line highlights an identified peptide in each sample, whereas a white line shows that no peptide was identified in that sample). The figure illustrates the data completeness.

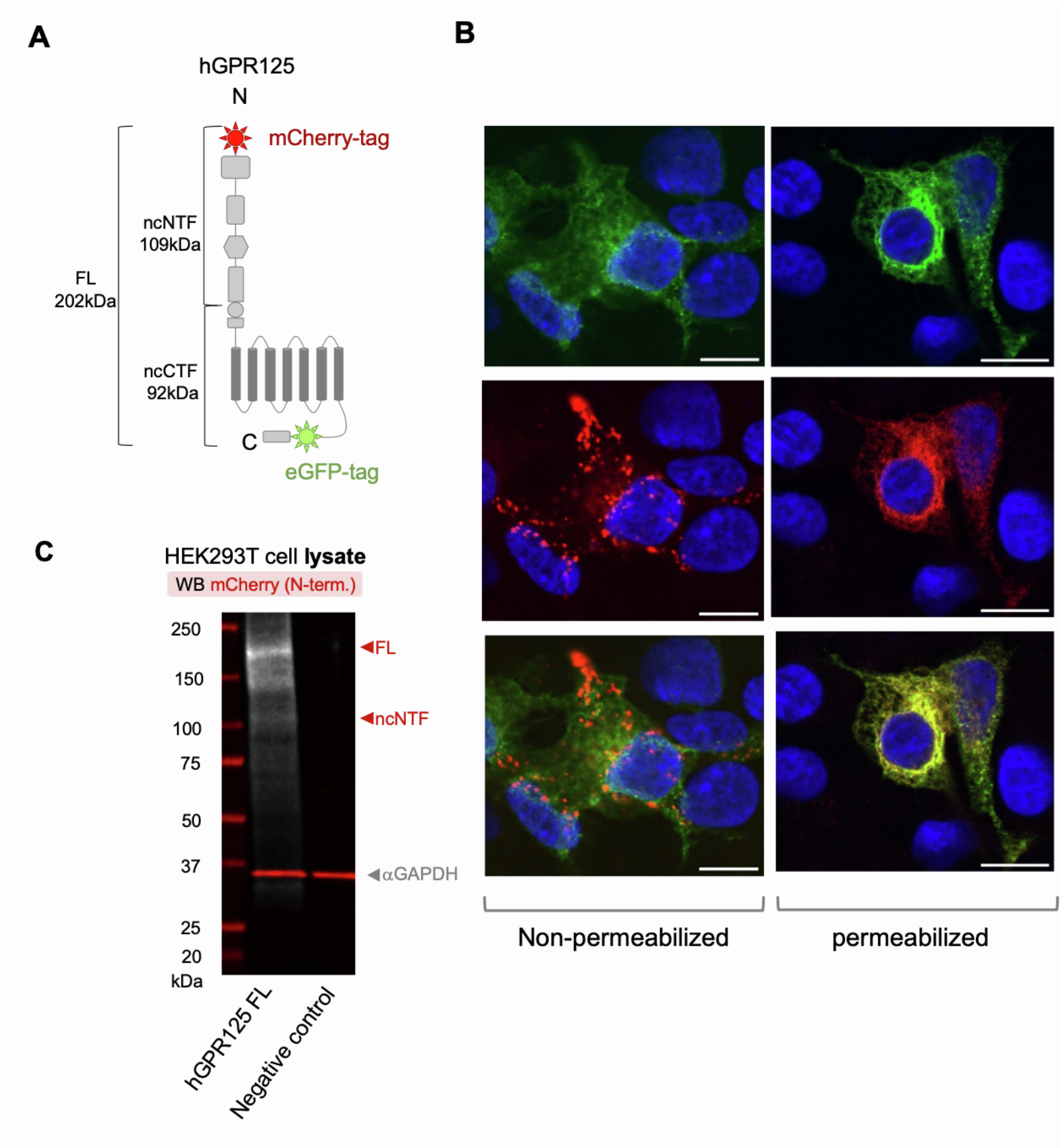

Figure S3. Permeabilization disrupts detection of intracellular vesicles harboring hGPR125 ncNTF. *Related to Figure 2.*

(A) Schematic overview of hGPR125 with an N-terminal mCherry-tag and a C-terminal eGFP-tag indicating the molecular sizes of the cleavage fragments.
(B) Immunofluorescent staining of transfected HEK293T cells expressing hGPR125 shown in (A) hGPR125 ncNTF and ncCTF can be found separately in HEK293T cells (left panels). Permeabilization destroys the intracellular vesicles harboring the hGPR125 ncNTF leading to a co-localization of the ncNTF and ncCTF (right panels). Width of scale bar is 10 µm.
(C) Immunoblot analysis of HEK293T cells expressing the fluorescently labeled GPR125 construct shown in (A)**.** The anti-mCherry detects the N-terminal tag of GPR125. The negative control shows cell lysates of cells expressing the empty vector (pcDNA3.1+). Anti-GAPDH has been included as a loading control. Arrows indicate the FL and the ncNTF.

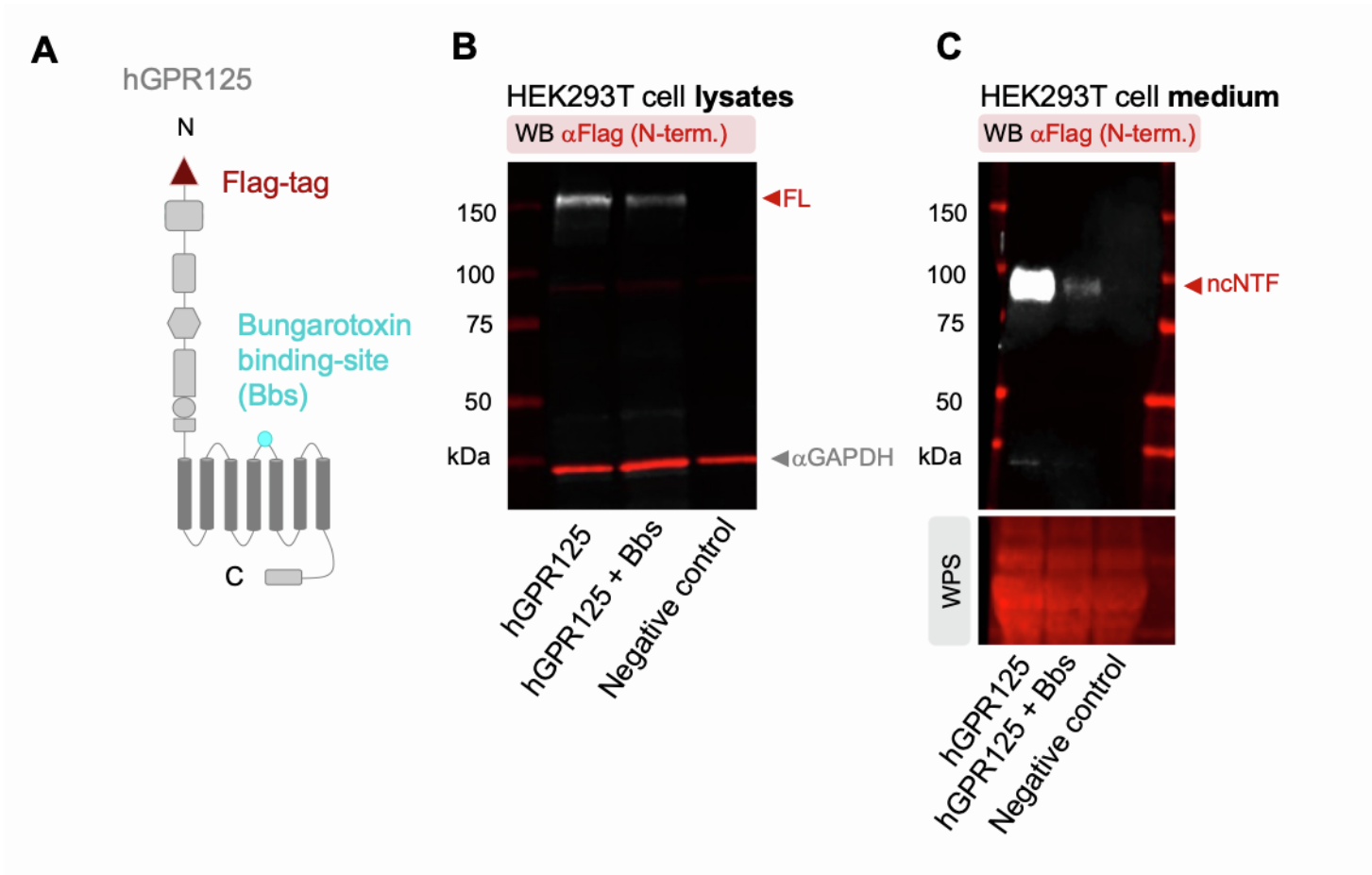

Figure S4. The GPR125 construct used in the live cell microscopy is cleaved and the ncNTF is released into the cell medium. *Related to Figure 2.*

(A-C) Control for hGPR125 live cell microscopy used in Figure 2B and C showing that this construct is cleaved and the ncNTF is released into the cell medium. **(A)** Schematic overview of hGPR125 showing the localization of the Flag-tag and the α-bungarotoxin binding site. **(B, C)** Immunoblot analysis of HEK293T cells and medium expressing hGPR125 used for the live cell microscopy in Figure 2B and C. The anti-Flag detects the N-terminal tag of GPR125. The negative control shows cell lysates of cells expressing the empty vector (pcDNA3.1+). Anti-GAPDH was included in (B) as a loading control and a whole protein stain (WPS) was performed in (C) as a loading control. Arrows indicate the FL and the cleavage fragments.

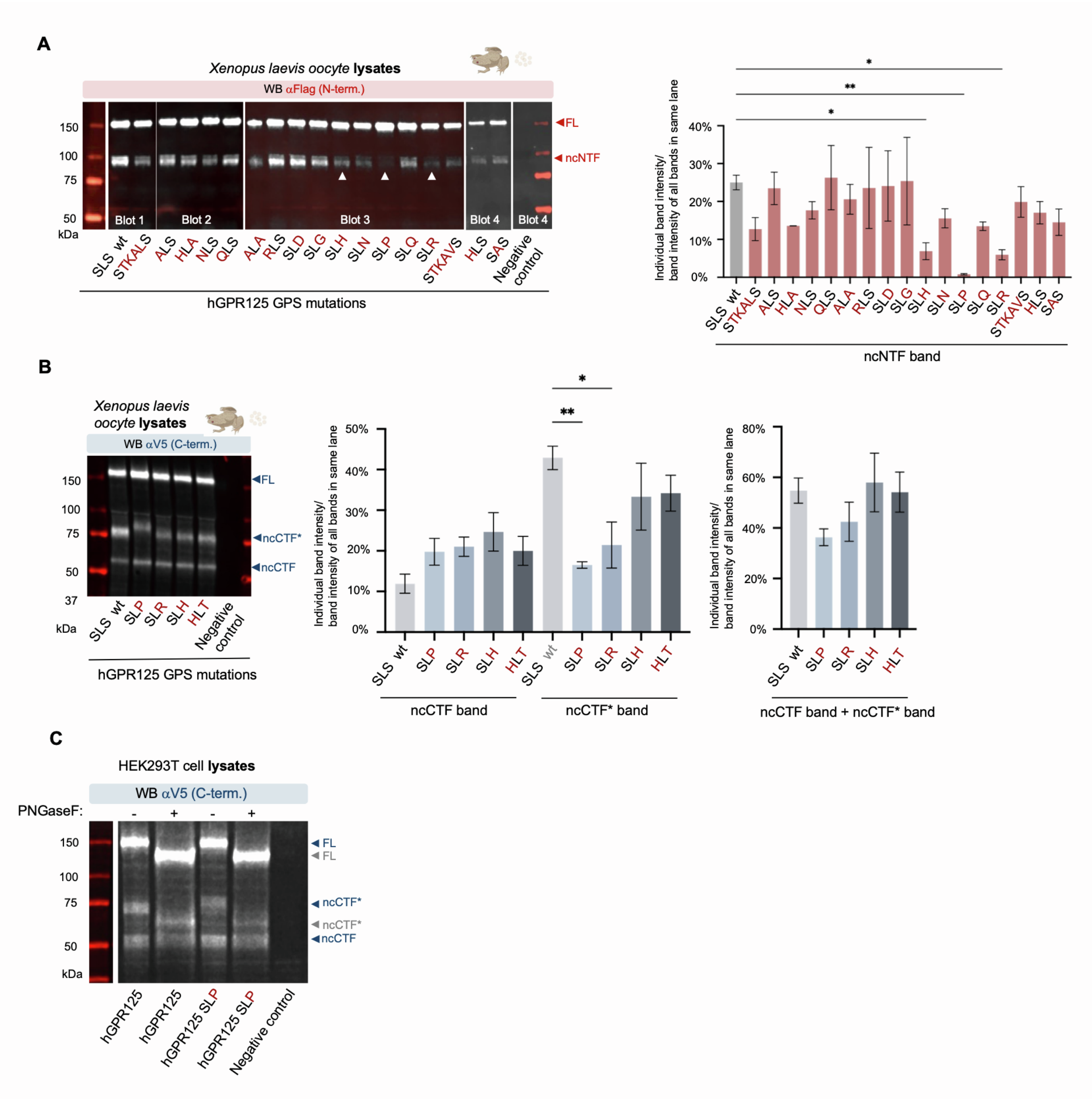

Figure S5. Mutations in the GPCR autoproteolysis site of hGPR125 affect the amount of ncNTF in the cell lysate fraction. *Related to Figure 3.*

(A and B) hGPR125 GPS mutations SLH, SLP and SLR show significantly less ncNTF (indicated with white arrowheads) in the cell lysate fraction of *Xenopus laevis* oocytes while the amount of ncCTF is less affected. (Left panels) Immunoblot analysis of *Xenopus laevis* oocyte lysates expressing hGPR125 wt and GPS mutants using the **(A)** anti-Flag targeting the N-terminal tag of hGPR125 and **(B)** anti-V5 targeting the C-terminal tag of hGPR125. The negative control shows cell lysates of cells expressing the empty vector (pXOOM). The anti-GAPDH was included as a loading control. (Right panels) Quantification of the GPR125 FL, ncNTF or ncCTF and ncCTF* protein bands. Red arrows indicate the FL and the cleavage fragments. Data are presented as mean ± SEM of at least n=3 independent experiments (n=12 for SLS in A); *$p < 0.05$, **$p < 0.01$ by one-way ANOVA followed by Dunnett's (A, B right) or Šídák's (B left) multiple-comparisons test compared to the wt. Non-significant p-values are not shown. Full statistical details are provided in Table S4.

(C) The ncCTF* of hGPR125 SLP mutant is more glycosylated than the ncCTF* of the wt. Immunoblot analysis of HEK293T cell lysates treated with PNGase F and expressing hGPR125 wt and the GPS mutant SLP. The anti-V5 recognizes the C-terminal tag of GPR125.

The negative control shows lysates of cells expressing the empty vector (pcDNA3.1+). Arrows indicate the FL and cleavage fragments with (gray arrow) and without (dark blue arrow) PNGase F.

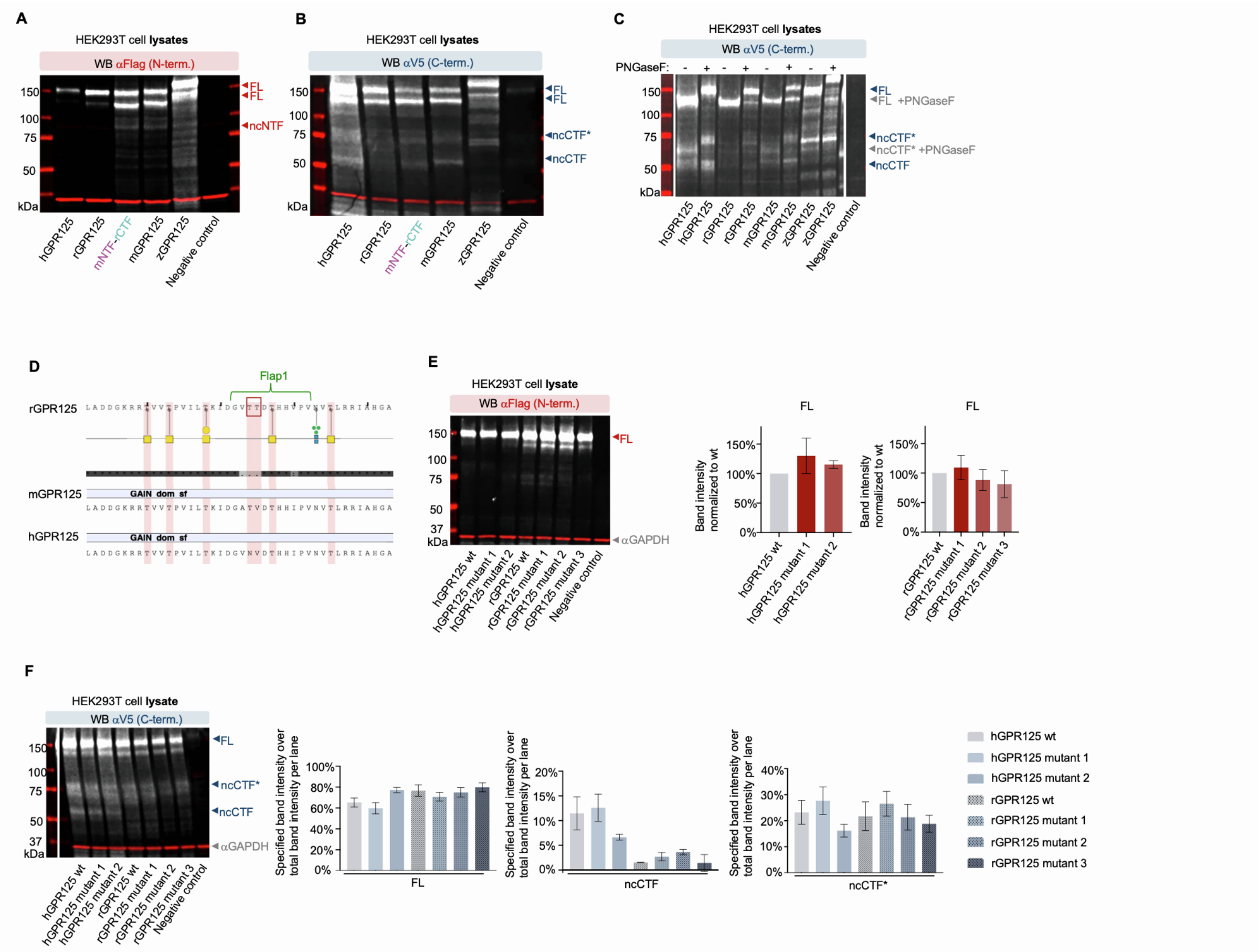

Figure S6. Mutations between the Flap1 and the GPCR autoproteolysis site do not significantly affect GPR125 cleavage. *Related to Figure 4.*

(A and B) The whole ncNTF of GPR125, not the GPS alone regulates cleavage. Western blots showing that the human, mouse, as well as the chimeric mouse-rat receptor are cleaved while the rat GPR125 is not. The anti-Flag antibody detects the N-terminal tag **(A)** and the anti-V5 antibody the C-terminal tag **(B)** of the GPR125 receptors. The negative control shows cell lysates of cells expressing the empty vector (pcDNA3.1+). The anti-GAPDH was included as a loading control. Arrows indicate the FL and the cleavage fragments.
(C) The human, mouse and rat GPR125 FL and ncCTF* are highly glycosylated. Immunoblot analysis of control and PNGase F treated HEK293T cells expressing zebrafish, mouse, rat and human GPR125. The anti-V5 recognizes the C-terminal tag of GPR125. Arrows indicate the FL and cleavage fragments with and without PNGase F.
(D) The rGPR125 has a Thr-Thr motif within the Flap1 which makes it a better candidate for O-glycosylation compared to the human and mouse receptor. Glycosylation prediction tool NetOGlyc-4.0 (1) showing the predicted O-glycosylation pattern around the Flap1 motif of the aligned human, mouse and rat receptor. The yellow squares indicate the experimentally validated O-glycosylation sites, and the blue-green symbol indicates an N-glycosylation site.
(E, F) Mutations within the human or rat GPR125 Flap1, or the region between the Flap1 and the GPS, do not significantly affect the amount of cleavage or receptor expression. (Left panels) Immunoblot analysis of HEK293T cell lysates expressing the human or rat wt receptor as well as the human or rat receptor carrying mutations between the Flap1 and GPS (see also Figure 4A for an overview of the mutations). The negative control shows cell lysates of cells expressing the empty vector (pcDNA3.1+). The anti-Flag antibody detects the N-terminal tag **(E)** and the anti-V5 antibody the C-terminal tag **(F)** of the GPR125 receptors. The anti-GAPDH was included as a loading control. (Right panels) Quantification of the GPR125 FL, ncCTF and ncCTF* protein bands. Arrows indicate the FL and the cleavage fragments. Data are

presented as mean ± SEM of n=3 independent experiments, analyzed by one-way ANOVA followed by Dunnett's (E) or Šídák's (F) multiple-comparisons test compared to the hGPR125 or rGPR125 wt. Non-significant p-values are not shown. Full statistical details are provided in Table S4.

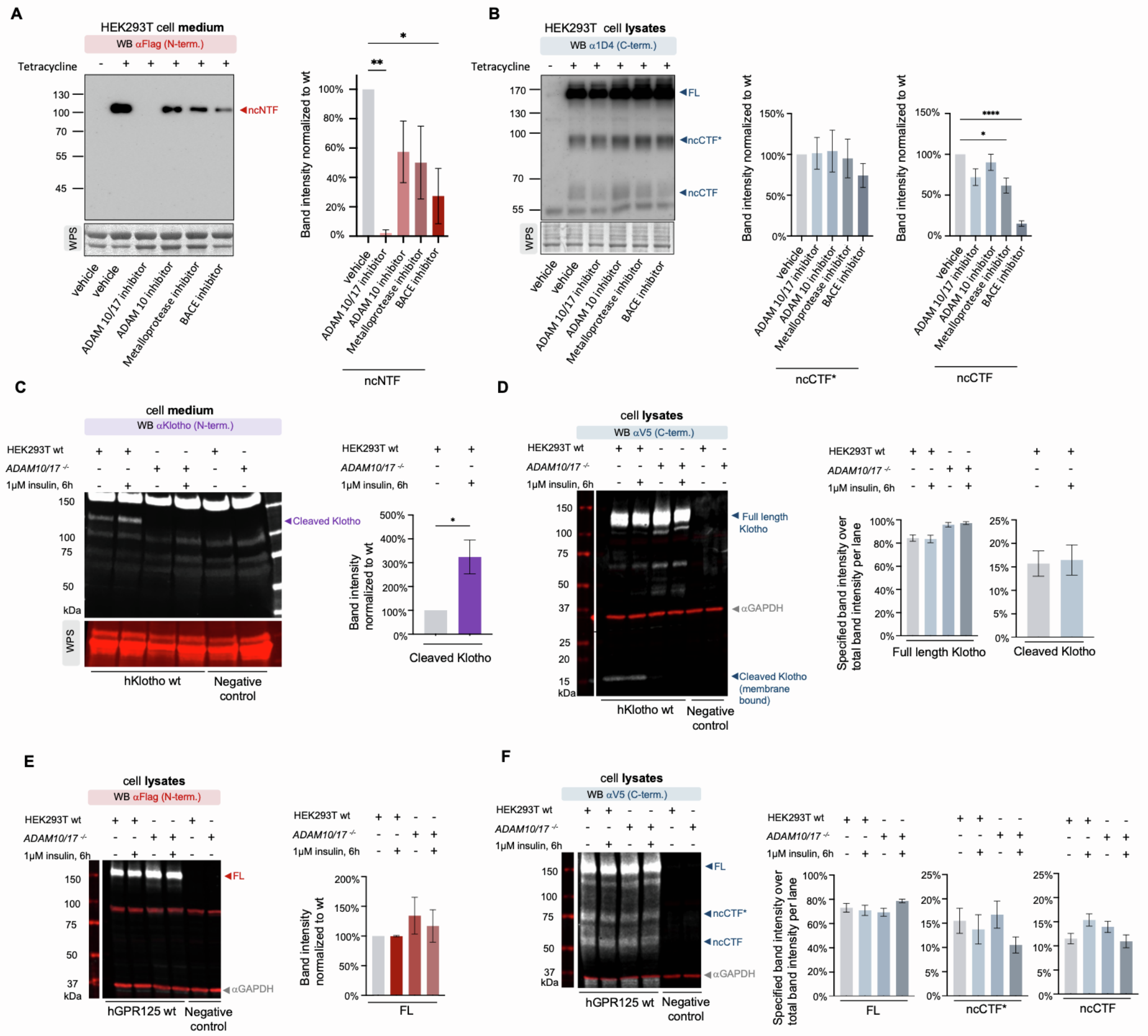

Figure S7. Involvement of ADAM10/17 and insulin in the cleavage and shedding of hKlotho and hGPR125. ***Related to Figure 4.***

(A, B) The ADAM10/17 inhibitor decreases the amount of shed GPR125 ncNTF but not the amount of ncCTF and ncCTF*. Vehicle (DMSO) ADAM10/17 inhibitor (GW280264X), ADAM10 inhibitor (GI254023X), metalloprotease inhibitor (batimastat), beta-secretase (BACE) inhibitor (0664283). **(A)** (Left) Immunoblot showing the amount of shed ncNTF in the cell medium of tetracycline-inducible GPR125 HEK293 cells in the presence of several protease inhibitors. The anti-Flag antibody detects the N-terminal tag of GPR125 receptor. A whole protein stain (WPS) was performed as a loading control. Arrows indicate the FL and the cleavage fragments. (Right) Quantification of the GPR125 ncNTF protein bands normalized to the wt. **(B)** (Left) Immunoblot showing the amount of FL, ncCTF and ncCTF* in cell lysates of tetracycline-inducible GPR125 HEK293 cells in the presence of several protease inhibitors. The anti-1D4 antibody detects the C-terminal tag of GPR125 receptor. A whole protein stain (WPS) was performed as a loading control. Arrows indicate the FL and the cleavage fragments. (Right) Quantification of the GPR125 ncCTF and ncCTF* protein bands normalized to the wt. Data are presented as mean ± SEM of n=4 independent experiments; *$p < 0.05$, **$p < 0.01$ and **** $p < 0.0001$ by one-way ANOVA followed by Dunnett's multiple-comparisons test compared to the vehicle control. Non-significant p-values are not shown. Full statistical details are provided in Table S4.

(C, D) Insulin stimulates the amount of shed Klotho in the presence of ADAM10/17. **(C)** (Left) Immunoblot showing the amount of cleaved Klotho in the cell medium in HEK293T wt and ADAM10/17$^{-/-}$ cells with and without the addition of insulin. The anti-Klotho antibody detects the cleaved Klotho. The negative control shows lysates of cells expressing the empty vector (pcDNA3.1+). A whole protein stain (WPS) was performed as a loading control. Arrows

indicate the cleaved Klotho. (Right) Quantification of the cleaved Klotho protein bands in the cell medium. Data are presented as mean ± SEM of n=3 independent experiments; *$p < 0.05$ by an unpaired t-test compared to the controls. Full statistical details are provided in Table S4. **(D)** (Left) Immunoblot showing the amount of full length and cleaved Klotho in HEK293T wt and ADAM10/17$^{-/-}$ cell lysates with and without the addition of insulin. The anti-V5 antibody detects the C-terminal tag of Klotho. The negative control shows lysates of cells expressing the empty vector (pcDNA3.1+). Anti-GAPDH was included as a loading control. Arrows indicate the full length and cleaved Klotho. (Right) Quantification of the full length and cleaved Klotho protein bands. Data are presented as mean ± SEM of n=3 independent experiments by an unpaired t-test (right) or by one-way ANOVA (left) followed by Šídák's multiple-comparisons test compared to the controls. Non-significant p-values are not shown. Full statistical details are provided in Table S4.

(E, F) Insulin stimulates the amount of shed GPR125 ncNTF and leads to a detectable ncNTF* in wt and ADAM10/17$^{-/-}$ cells. **(E)** (Left) Immunoblot showing the amount FL and cleavage fragments in lysates of HEK293T wt and ADAM10/17$^{-/-}$ cells with and without the addition of insulin. The anti-Flag antibody detects the N-terminal tag of GPR125. The negative control shows cell lysates of cells expressing the empty vector (pcDNA3.1+). Anti-GAPDH was included as a loading control. Arrows indicate the GPR125 FL. (Right) Quantification of the GPR125 FL protein bands. **(F)** (Left) Immunoblot showing the amount FL and cleavage fragments in cell lysates of HEK293T wt and ADAM10/17$^{-/-}$ cells with and without the addition of insulin. The anti-ncCTF antibody detects the C-terminal tag of GPR125. The negative control shows cell lysates of cells expressing the empty vector (pcDNA3.1+). Anti-GAPDH was included as a loading control. Arrows indicate the GPR125 FL, ncCTF and ncCTF*. (Right) Quantification of the GPR125 FL, ncCTF and ncCTF* protein bands. Data are presented as mean ± SEM of n=3 independent experiments, analyzed by one-way ANOVA followed by Dunnett's (E) or Šídák's (F) multiple-comparisons test compared to the controls. Non-significant p-values are not shown. Full statistical details are provided in Table S4.

Trypsin and TrypN cleavage sites:
Trypsin cleaves C-term of R/K and TrypN cleaves N-term of **R/K**

MEPPGRRRGRAQPPLLLPLSLLALLALLGGGGGGGAAALPAGCKHDGRPRGAGRAAGAAEGKVVCS
SLELAQVLPPDTLPNRTVTLILSNNKISELKNGSFSGLSLLERLDLRNNLISSIDPGAFWGLSSLKRLDLT
NNRIGCLNADIFRGLTNLVRLNLSGNLFSSLSQGTFDYLASLRSLEFQTEYLLCDCNILWMHRWVKEKN
ITVRDTRCVYPKSLQAQPVTGVKQELLTCDPPLELPSFYMTPSHRQVVFEGDSLPFQCMASYIDQDM
QVLWYQDGRIVETDESQGIFVEKNMIHNCSLIASALTISNIQAGSTGNWGCHVQTKRGNNTRTVDIVVL
ESSAQYCPPERVVNNKGDFRWPRTLAGITAYLQCTRNTHGSGIYPGNPQDERKAWRRCDRGGFWA
DDDYSRCQYANDVTRVLYMFNQMPLNLTNAVATARQLLAYTVEAANFSDKMDVIFVAEMIEKFGRFTK
EEKSKELGDVMVDIASNIMLADERVLWLAQREAKACSRIVQCLQRIATYRLAGGAHVYSTYSPNIALEA
YVIKSTGFTGMTCTVFQKVAASDRTGLSDYGRRDPEGNLDKQLSFKCNVSNTFSSLALKNTIVEASIQL
PPSLFSPKQKRELRPTDDSLYKLQLIAFRNGKLFPATGNSTNLADDGKRRTVVTPVILTKIDGVNVDTH
HIPVNVTLRRIAHGADAVAARWDFDLLNGQGGWKSDGCHILYSDENITTIQCYSLSNYAVLMDLTGSEL
YTQAASLLHPVVYTTAIILLLCLLAVIVSYIYHHSLIRISLKSWHMLVNLCFHIFLTCVVFVGGITQTRNASI
CQAVGIILHYSTLATVLWVGVTARNIYKQVTKKAKRCQDPDEPPPPPRPMLRFYLIGGGIPIIVCGITAA
ANIKNYGSRPNAPYCWMAWEPSLGAFYGPASFITFVNCMYFLSIFIQLKRHPERKYELKEPTEEQQRL
AANENGEINHQDSMSLSLISTSALENEHTFHSQLLGASLTLLLYVALWMFGALAVSLYYPLDLVFSFVF
GATSLSFSAFFVVHHCVNREDVRLAWIMTCCPGRSSYSVQVNVQPPNSNGTNGEAPKCPNSSAESS
CTNKSASSFKNSSQGCKLTNLQAAAAQCHANSLPLNSTPQLDNSLTEHSMDNDIKMHVAPLEVQFRT
NVHSSRHHKNRSKGHRASRLTVLREYAYDVPTSVEGSVQNGLPKSRLGNNEGHSRSRRAYLAYRER
QYNPPQQDSSDACSTLPKSSRNFEKPVSTTSKKDALRKPAVVELENQQKSYGLNLAIQNGPIKSNGQ
EGPLLGTDSTGNVRTGLWKHETTV

Figure S8: Predicted Trypsin and TrypN cleavage sites in GPR125. ***Related to Figure 5***
Protein sequence of GPR125 indicating Trypsin and TrypN cleavage sites (green). There are no Trypsin and TrypN cleavage sites in close proximity to the GPS (highlighted in gray).

Table S1. Predicted changes to protein stability and solvent accessible surface area for hGPR125 GPCR autoproteolysis site mutations. *Related to Figure 3.*

Residue	Original	Mutated	Motif	Δ Stability (solvated)	Δ Hydropathy	Δ SASA (polar)	Δ SASA (non polar)	Δ SASA (total)	Δ Prime Energy	ΔE Covalent binding	ΔE van-der-Waals	ΔE Coulomb	ΔE Generalized Born electrostatic solvation	ΔE Stability Lipophilic interactions	ΔE Hydrogen bonding	ΔE π-π correction	ΔE Self-contact correction
**A:738**	SER	ARG	SLR	-10,36	-0,04	-23,13	-20,8	-44,7	-36,22	14,56	-9,51	24,34	-58,6	-6,8	-2,37	0,15	1,99
**A:738**	SER	HSD	SLH	-1,81	0,01	-4,08	-2,83	-9,07	-7,37	14,07	-3,02	-21,32	5,8	-3,87	0,12	-1,21	2,05
**A:738**	SER	HSE	SLH	4,85	-0,03	-6,3	-8,31	-15,33	-2,52	17,88	-2,09	-22,46	8,23	-4,66	-0,88	-0,41	1,87
**A:738**	SER	HSP	SLH	0,03	-0,06	-1,93	-7,66	-11,5	-3,81	19,45	-5,53	-0,98	-12,45	-2,76	-2,46	-0,78	1,7
**A:738**	SER	PRO	SLP	41,91	0,03	-14,23	11,65	-2,7	76,34	27,78	20,43	24,78	6,62	-7,64	1,06	-0,72	4,02
**A:738**	SER	THR	SLT	4,99	0,19	-3,46	-2,42	-7,1	2,97	10,48	-4,26	-2,41	1,43	-1,43	-0,37	0,27	-0,74
**A:736**	SER	HSD	HLS	-1,05	-0,43	20,31	-3,94	-22,74	-6,99	7,56	-4,66	3,71	-8,76	-4,83	-2,11	-2,12	4,23
**A:736**	SER	HSE	HLS	11,36	-0,12	-2,13	-3,98	-15,37	4,97	15,47	1,15	-5,91	-4,57	-2	1,6	-4,59	3,82
**A:736**	SER	HSP	HLS	-7,35	-0,61	39,24	-11,18	3,96	-13,27	18,38	-4,84	1,51	-21,18	-4,1	-5,61	-1,43	4
**A:736 A:738**	SER SER	HSD THR	HLT	2,22	-0,71	51,19	-5,95	19,47	-5,91	15,57	5,77	-2,07	-14,54	-6,28	-1,97	-4,08	1,7
**A:736 A:738**	SER SER	HSE THR	HLT	8,02	-0,18	2,57	-3,9	-9,85	-1	18,62	2,31	-11,61	-3,97	-5,17	1,83	-4,54	1,54
**A:736 A:738**	SER SER	HSP THR	HLT	-5,08	-0,2	9,99	-7,83	1,03	-13,08	13,18	2,54	-3,31	-16,3	-4,61	-2,89	-3,48	1,78
**A:736**	SER	ALA	ALS	4,56	0	3,66	1,37	9,02	5,1	0,44	-0,96	5,56	-6,71	2,35	0,28	0,05	4,09
**A:737**	LEU	ALA	SAS	14,64	-0,01	0,38	12,02	13,58	7,56	-3,23	3,43	-3,8	-3,17	14,18	-0,14	0,19	0,1
**A:738**	SER	ALA	SLA	2,64	0,04	-4,68	7,2	1,73	3,63	5,27	-1,49	-4,04	0,27	0,39	0,74	-0,06	2,55
**A:736 A:737**	SER LEU	ALA ALA	AAS	16,33	0,06	-7,23	9,45	2,87	10,03	-2,52	3,8	-5,19	-3,97	12,69	0,83	0,29	4,1
**A:736 A:738**	SER SER	ALA ALA	ALA	4,78	0,05	-5,71	8,23	0,61	6,45	5,15	-2,43	-3,12	-0,77	0,27	0,87	-0,06	6,53
**A:737 A:738**	LEU SER	ALA ALA	SAA	18,5	0	-3,97	16,84	14,55	12,44	2,12	3,96	-9,24	-2,55	14,03	1,65	0,08	2,4
**A:736 A:737 A:738**	SER LEU SER	ALA ALA ALA	AAA	19,52	0,06	-7,65	17,98	8,12	14,24	1,91	3,35	-8,39	-4,19	13,39	1,58	0,2	6,39

Table S2. List of all identified Trypsin and TrypN peptides of GPR125. *Related to Figure 5.*

Peptide Sequence	Modified Sequence	Prev AA	Next AA	Peptide type	Enzyme	Start	End	Peptide Length	Protein	Identified in:
**LLGGGGGDY**	LLGGGGGDY	A	K	Nterm	TrypN	19	27	9	sp|Q8IWK6|AGRA3_HUMAN	Medium
**GGAAALPAGCK**	GGAAALPAGC[57.0215]K	K	H	Internal	Trypsin	34	44	11	sp|Q8IWK6|AGRA3_HUMAN	Medium
**RTVTLILSNN**	RTVTLILSNN	N	K	Internal	TrypN	82	91	10	sp|Q8IWK6|AGRA3_HUMAN	Lysate
**TVTLILSN**	TVTLILSN	R	N	Cterm	Trypsin	83	90	8	sp|Q8IWK6|AGRA3_HUMAN	Medium
**TVTLILSNN**	TVTLILSNN	R	K	Cterm	Trypsin	83	91	9	sp|Q8IWK6|AGRA3_HUMAN	Medium
**TVTLILSNNK**	TVTLILSNNK	R	I	Internal	Trypsin	83	92	10	sp|Q8IWK6|AGRA3_HUMAN	Medium and Lysate
**TLILSNNK**	TLILSNNK	V	I	Internal	Trypsin	85	92	8	sp|Q8IWK6|AGRA3_HUMAN	Medium
**NGSFSGLSLLER**	NGSFSGLSLLER	K	L	Internal	Trypsin	98	109	12	sp|Q8IWK6|AGRA3_HUMAN	Medium
**GSFSGLSLLE**	GSFSGLSLLE	N	R	Nterm	TrypN	99	108	10	sp|Q8IWK6|AGRA3_HUMAN	Medium
**SFSGLSLLE**	SFSGLSLLE	G	R	Nterm	TrypN	100	108	9	sp|Q8IWK6|AGRA3_HUMAN	Medium
**FSGLSLLER**	FSGLSLLER	S	L	Internal	Trypsin	101	109	9	sp|Q8IWK6|AGRA3_HUMAN	Medium
**NNLISSIDPGAF**	NNLISSIDPGAF	R	W	Cterm	Trypsin	114	125	12	sp|Q8IWK6|AGRA3_HUMAN	Medium
**NNLISSIDPGAFWGLSSLK**	NNLISSIDPGAFWGLSSLK	R	R	Internal	Trypsin	114	132	19	sp|Q8IWK6|AGRA3_HUMAN	Medium
**PGAFWGLSSLK**	PGAFWGLSSLK	D	R	Internal	Trypsin	122	132	11	sp|Q8IWK6|AGRA3_HUMAN	Medium
**RLDLTNN**	RLDLTNN	K	R	Internal	TrypN	133	139	7	sp|Q8IWK6|AGRA3_HUMAN	Medium
**RIGCLNADIF**	RIGC[57.0215]LNADIF	N	R	Internal	TrypN	140	149	10	sp|Q8IWK6|AGRA3_HUMAN	Medium
**IGCLNADIFR**	IGC[57.0215]LNADIFR	R	G	Internal	Trypsin	141	150	10	sp|Q8IWK6|AGRA3_HUMAN	Medium
**CLNADIFR**	C[57.0215]LNADIFR	G	G	Internal	Trypsin	143	150	8	sp|Q8IWK6|AGRA3_HUMAN	Medium
**RGLTNLV**	RGLTNLV	F	R	Internal	TrypN	150	156	7	sp|Q8IWK6|AGRA3_HUMAN	Medium
**GLTNLVR**	GLTNLVR	R	L	Internal	Trypsin	151	157	7	sp|Q8IWK6|AGRA3_HUMAN	Medium
**LNLSGNLF**	LNLSGNLF	R	S	Cterm	Trypsin	158	165	8	sp|Q8IWK6|AGRA3_HUMAN	Medium
**SLEFQTEY**	SLEFQTEY	R	L	Cterm	Trypsin	181	188	8	sp|Q8IWK6|AGRA3_HUMAN	Medium
**KSLQAQPVTGV**	KSLQAQPVTGV	P	K	Internal	TrypN	218	228	11	sp|Q8IWK6|AGRA3_HUMAN	Medium
**SLQAQPVTGVK**	SLQAQPVTGVK	K	Q	Internal	Trypsin	219	229	11	sp|Q8IWK6|AGRA3_HUMAN	Medium and Lysate
**SLQAQPVTGVK**	SLQAQPVTGVK	K	Q	Internal	Trypsin	219	229	11	sp|Q8IWK6|AGRA3_HUMAN	Medium and Lysate
**QAQPVTGVK**	QAQPVTGVK	L	Q	Internal	Trypsin	221	229	9	sp|Q8IWK6|AGRA3_HUMAN	Medium
**AQPVTGVK**	AQPVTGVK	Q	Q	Internal	Trypsin	222	229	8	sp|Q8IWK6|AGRA3_HUMAN	Medium
**KQELLTCDPPLELPSFYMTPSH**	KQELLTC[57.0215]DPPLELPSFYMTPSH	V	R	Internal	TrypN	229	250	22	sp|Q8IWK6|AGRA3_HUMAN	Medium
**QELLTCDPPLELPSFYMTPSHR**	QELLTC[57.0215]DPPLELPSFYMTPSHR	K	Q	Internal	Trypsin	230	251	22	sp|Q8IWK6|AGRA3_HUMAN	Medium
**PPLELPSFYMTPSH**	PPLELPSFYMTPSH	D	R	Nterm	TrypN	237	250	14	sp|Q8IWK6|AGRA3_HUMAN	Medium
**PPLELPSFYMTPSH**	PPLELPSFYM[15.9949]TPSH	D	R	Nterm	TrypN	237	250	14	sp|Q8IWK6|AGRA3_HUMAN	Medium
**PPLELPSFYMTPSHR**	PPLELPSFYMTPSHR	D	Q	Internal	Trypsin	237	251	15	sp|Q8IWK6|AGRA3_HUMAN	Medium
**PSFYMTPSH**	PSFYMTPSH	L	R	Nterm	TrypN	242	250	9	sp|Q8IWK6|AGRA3_HUMAN	Medium
**ASYIDQDMQVLWYQDGR**	ASYIDQDMQVLWYQDGR	M	I	Internal	Trypsin	266	282	17	sp|Q8IWK6|AGRA3_HUMAN	Medium

**RIVETDESQGIFVE**	RIVETDESQGIFVE	G	K	Internal	TrypN	282	295	14	sp|Q8IWK6|AGRA3_HUMAN	Medium and Lysate
**IVETDESQGIFVEK**	IVETDESQGIFVEK	R	N	Internal	Trypsin	283	296	14	sp|Q8IWK6|AGRA3_HUMAN	Medium and Lysate
**ETDESQGIFVEK**	ETDESQGIFVEK	V	N	Internal	Trypsin	285	296	12	sp|Q8IWK6|AGRA3_HUMAN	Medium
**ESQGIFVEK**	ESQGIFVEK	D	N	Internal	Trypsin	288	296	9	sp|Q8IWK6|AGRA3_HUMAN	Medium
**SQGIFVEK**	SQGIFVEK	E	N	Internal	Trypsin	289	296	8	sp|Q8IWK6|AGRA3_HUMAN	Medium
**RTVDIVVLESSAQYCPPE**	RTVDIVVLESSAQYC[57.0215]PPE	T	R	Internal	TrypN	335	352	18	sp|Q8IWK6|AGRA3_HUMAN	Medium
**TVDIVVLE**	TVDIVVLE	R	S	Cterm	Trypsin	336	343	8	sp|Q8IWK6|AGRA3_HUMAN	Medium
**TVDIVVLESSAQYCPPER**	TVDIVVLESSAQYC[57.0215]PPER	R	V	Internal	Trypsin	336	353	18	sp|Q8IWK6|AGRA3_HUMAN	Medium
**SSAQYCPPE**	SSAQYC[57.0215]PPE	E	R	Nterm	TrypN	344	352	9	sp|Q8IWK6|AGRA3_HUMAN	Medium
**RTLAGITAYLQCT**	RTLAGITAYLQC[57.0215]T	P	R	Internal	TrypN	365	377	13	sp|Q8IWK6|AGRA3_HUMAN	Medium
**TLAGITAY**	TLAGITAY	R	L	Cterm	Trypsin	366	373	8	sp|Q8IWK6|AGRA3_HUMAN	Medium and Lysate
**TLAGITAYL**	TLAGITAYL	R	Q	Cterm	Trypsin	366	374	9	sp|Q8IWK6|AGRA3_HUMAN	Medium
**TLAGITAYLQ**	TLAGITAYLQ	R	C	Cterm	Trypsin	366	375	10	sp|Q8IWK6|AGRA3_HUMAN	Medium
**RNTHGSGIYPGNPQDE**	RNTHGSGIYPGNPQDE	T	R	Internal	TrypN	378	393	16	sp|Q8IWK6|AGRA3_HUMAN	Medium and Lysate
**RNTHGSGIYPGNPQDE**	RNTHGSGIYPGNPQDE	T	R	Internal	TrypN	378	393	16	sp|Q8IWK6|AGRA3_HUMAN	Medium and Lysate
**NTHGSGIYPGNPQDER**	NTHGSGIYPGNPQDER	R	K	Internal	Trypsin	379	394	16	sp|Q8IWK6|AGRA3_HUMAN	Medium and Lysate
**YPGNPQDE**	YPGNPQDE	I	R	Nterm	TrypN	386	393	8	sp|Q8IWK6|AGRA3_HUMAN	Medium
**PGNPQDE**	PGNPQDE	Y	R	Nterm	TrypN	387	393	7	sp|Q8IWK6|AGRA3_HUMAN	Medium
**PGNPQDER**	PGNPQDER	Y	K	Internal	Trypsin	387	394	8	sp|Q8IWK6|AGRA3_HUMAN	Medium
**RGGFWADDDYS**	RGGFWADDDYS	D	R	Internal	TrypN	402	412	11	sp|Q8IWK6|AGRA3_HUMAN	Medium and Lysate
**RGGFWADDDYS**	RGGFWADDDYS	D	R	Internal	TrypN	402	412	11	sp|Q8IWK6|AGRA3_HUMAN	Medium and Lysate
**GGFWADDDYSR**	GGFWADDDYSR	R	C	Internal	Trypsin	403	413	11	sp|Q8IWK6|AGRA3_HUMAN	Medium and Lysate
**GFWADDDYS**	GFWADDDYS	G	R	Nterm	TrypN	404	412	9	sp|Q8IWK6|AGRA3_HUMAN	Medium
**WADDDYSR**	WADDDYSR	F	C	Internal	Trypsin	406	413	8	sp|Q8IWK6|AGRA3_HUMAN	Medium
**ADDDYSR**	ADDDYSR	W	C	Internal	Trypsin	407	413	7	sp|Q8IWK6|AGRA3_HUMAN	Medium
**RCQYANDVT**	RC[57.0215]QYANDVT	S	R	Internal	TrypN	413	421	9	sp|Q8IWK6|AGRA3_HUMAN	Medium
**CQYANDVTR**	C[57.0215]QYANDVTR	R	V	Internal	Trypsin	414	422	9	sp|Q8IWK6|AGRA3_HUMAN	Medium
**YANDVTR**	YANDVTR	Q	V	Internal	Trypsin	416	422	7	sp|Q8IWK6|AGRA3_HUMAN	Medium
**RVLYMFNQMPLNLTNAVATA**	RVLYMFNQMPLNLTNAVATA	T	R	Internal	TrypN	422	441	20	sp|Q8IWK6|AGRA3_HUMAN	Medium
**VLYMFNQMPLNLTNAVATAR**	VLYMFNQMPLNLTNAVATAR	R	Q	Internal	Trypsin	423	442	20	sp|Q8IWK6|AGRA3_HUMAN	Medium
**RQLLAYTVEAANFSD**	RQLLAYTVEAANFSD	A	K	Internal	TrypN	442	456	15	sp|Q8IWK6|AGRA3_HUMAN	Lysate
**QLLAYTVEAANFSDK**	QLLAYTVEAANFSDK	R	M	Internal	Trypsin	443	457	15	sp|Q8IWK6|AGRA3_HUMAN	Medium and Lysate
**TVEAANFSDK**	TVEAANFSDK	Y	M	Internal	Trypsin	448	457	10	sp|Q8IWK6|AGRA3_HUMAN	Medium
**EAANFSDK**	EAANFSDK	V	M	Internal	Trypsin	450	457	8	sp|Q8IWK6|AGRA3_HUMAN	Medium
**MDVIFVAEMIEK**	MDVIFVAEMIEK	K	F	Internal	Trypsin	458	469	12	sp|Q8IWK6|AGRA3_HUMAN	Medium
**ELGDVMVDIASNIMLADER**	ELGDVMVDIASNIMLADER	K	V	Internal	Trypsin	481	499	19	sp|Q8IWK6|AGRA3_HUMAN	Medium
**SNIMLADE**	SNIMLADE	A	R	Nterm	TrypN	491	498	8	sp|Q8IWK6|AGRA3_HUMAN	Lysate
**RVLWLAQ**	RVLWLAQ	E	R	Internal	TrypN	499	505	7	sp|Q8IWK6|AGRA3_HUMAN	Medium and Lysate
**VLWLAQR**	VLWLAQR	R	E	Internal	Trypsin	500	506	7	sp|Q8IWK6|AGRA3_HUMAN	Medium and Lysate
**LAGGAHVYSTYSPN**	LAGGAHVYSTYSPN	R	I	Cterm	Trypsin	526	539	14	sp|Q8IWK6|AGRA3_HUMAN	Medium

**LAGGAHVYSTYSPNIALEAY**	LAGGAHVYSTYSPNIALEAY	R	V	Cterm	Trypsin	526	545	20	sp|Q8IWK6|AGRA3_HUMAN	Medium
**LAGGAHVYSTYSPNIALEAYVIK**	LAGGAHVYSTYSPNIALEAYVIK	R	S	Internal	Trypsin	526	548	23	sp|Q8IWK6|AGRA3_HUMAN	Medium and Lysate
**VYSTYSPNIALEAYVIK**	VYSTYSPNIALEAYVIK	H	S	Internal	Trypsin	532	548	17	sp|Q8IWK6|AGRA3_HUMAN	Medium
**STYSPNIALEAYVIK**	STYSPNIALEAYVIK	Y	S	Internal	Trypsin	534	548	15	sp|Q8IWK6|AGRA3_HUMAN	Medium
**IALEAYVIK**	IALEAYVIK	N	S	Internal	Trypsin	540	548	9	sp|Q8IWK6|AGRA3_HUMAN	Medium
**ALEAYVIK**	ALEAYVIK	I	S	Internal	Trypsin	541	548	8	sp|Q8IWK6|AGRA3_HUMAN	Medium
**STGFTGMTCTVFQK**	STGFTGMTC[57.0215]TVFQK	K	V	Internal	Trypsin	549	562	14	sp|Q8IWK6|AGRA3_HUMAN	Medium
**RTGLSDYG**	RTGLSDYG	D	R	Internal	TrypN	568	575	8	sp|Q8IWK6|AGRA3_HUMAN	Medium
**TGLSDYGR**	TGLSDYGR	R	R	Internal	Trypsin	569	576	8	sp|Q8IWK6|AGRA3_HUMAN	Medium
**RDPEGNLD**	RDPEGNLD	R	K	Internal	TrypN	577	584	8	sp|Q8IWK6|AGRA3_HUMAN	Medium
**DPEGNLDK**	DPEGNLDK	R	Q	Internal	Trypsin	578	585	8	sp|Q8IWK6|AGRA3_HUMAN	Medium
**CNVSNTFSSLALK**	C[57.0215]NVSNTFSSLALK	K	N	Internal	Trypsin	591	603	13	sp|Q8IWK6|AGRA3_HUMAN	Medium
**KNTIVEASIQLPPSLFSP**	KNTIVEASIQLPPSLFSP	L	K	Internal	TrypN	603	620	18	sp|Q8IWK6|AGRA3_HUMAN	Medium and Lysate
**NTIVEASIQLPPSLFSPK**	NTIVEASIQLPPSLFSPK	K	Q	Internal	Trypsin	604	621	18	sp|Q8IWK6|AGRA3_HUMAN	Medium and Lysate
**PPSLFSPK**	PPSLFSPK	L	Q	Internal	Trypsin	614	621	8	sp|Q8IWK6|AGRA3_HUMAN	Medium
**KLQLIAF**	KLQLIAF	Y	R	Internal	TrypN	635	641	7	sp|Q8IWK6|AGRA3_HUMAN	Medium and Lysate
**TVVTPVILTK**	TVVTPVILTK	R	I	Internal	Trypsin	664	673	10	sp|Q8IWK6|AGRA3_HUMAN	Medium and Lysate
**VTPVILTK**	VTPVILTK	V	I	Internal	Trypsin	666	673	8	sp|Q8IWK6|AGRA3_HUMAN	Medium
**IAHGADAVAAR**	IAHGADAVAAR	R	W	Internal	Trypsin	693	703	11	sp|Q8IWK6|AGRA3_HUMAN	Medium
**WDFDLLNGQGGWK**	WDFDLLNGQGGWK	R	S	Internal	Trypsin	704	716	13	sp|Q8IWK6|AGRA3_HUMAN	Medium and Lysate
**SSYSVQVNVQPPNSNGTNGEAPK**	SSYSVQVNVQPPNSNGTNGEAPK	R	C	Internal	Trypsin	1066	1088	23	sp|Q8IWK6|AGRA3_HUMAN	Lysate
**KMHVAPLEVQF**	KMHVAPLEVQF	I	R	Internal	TrypN	1152	1162	11	sp|Q8IWK6|AGRA3_HUMAN	Lysate
**MHVAPLEVQFR**	MHVAPLEVQFR	K	T	Internal	Trypsin	1153	1163	11	sp|Q8IWK6|AGRA3_HUMAN	Lysate
**VAPLEVQF**	VAPLEVQF	H	R	Nterm	TrypN	1155	1162	8	sp|Q8IWK6|AGRA3_HUMAN	Lysate
**EYAYDVPTSVEGSVQNGLPK**	EYAYDVPTSVEGSVQNGLPK	R	S	Internal	Trypsin	1189	1208	20	sp|Q8IWK6|AGRA3_HUMAN	Medium and Lysate
**KPAVVELENQQ**	KPAVVELENQQ	R	K	Internal	TrypN	1268	1278	11	sp|Q8IWK6|AGRA3_HUMAN	Lysate
**SYGLNLAIQNGPIK**	SYGLNLAIQNGPIK	K	S	Internal	Trypsin	1280	1293	14	sp|Q8IWK6|AGRA3_HUMAN	Lysate
**SNGQEGPLLGTDSTGNVR**	SNGQEGPLLGTDSTGNVR	K	T	Internal	Trypsin	1294	1311	18	sp|Q8IWK6|AGRA3_HUMAN	Lysate

Table S3. List of all identified GluC peptides of GPR125 including missed cleavages and peptidoforms. *Related to Figure 5.*

Peptide Sequence	Modified Sequence	Start	End	Peptide Length	Protein
**ASIQLPPSLFSPKQ**	ASIQLPPSLFSPKQ	609	622	14	sp|Q8IWK6|AGRA3_HUMAN
**GKVVCSSLE**	GKVVC[57.0215]SSLE	61	69	9	sp|Q8IWK6|AGRA3_HUMAN
**LAQVLPPDTLPNRTVTLILSNNKISE**	LAQVLPPDTLPNRTVTLILSNNKISE	70	95	26	sp|Q8IWK6|AGRA3_HUMAN
**LLTCDPPLELPSFYMTPSHRQVVFE**	LLTC[57.0215]DPPLELPSFYMTPSHRQVVFE	232	256	25	sp|Q8IWK6|AGRA3_HUMAN
**LLTCDPPLELPSFYMTPSHRQVVFE**	LLTC[57.0215]DPPLELPSFYM[15.9949]TPSHRQVVFE	232	256	25	sp|Q8IWK6|AGRA3_HUMAN
**PPLELPSFYMTPSHRQVVFE**	PPLELPSFYMTPSHRQVVFE	237	256	20	sp|Q8IWK6|AGRA3_HUMAN
**SQGIFVE**	SQGIFVE	289	295	7	sp|Q8IWK6|AGRA3_HUMAN

Reference list

1. Steentoft C, Vakhrushev SY, Joshi HJ, Kong Y, Vester-Christensen MB, Schjoldager KT, et al. Precision mapping of the human O-GalNAc glycoproteome through SimpleCell technology. Embo j. 2013;32(10):1478-88.
